# Effectiveness of Seasonal Malaria Chemoprevention in Children under Ten Years of Age in Senegal: A Stepped-Wedge Cluster-Randomised Trial

**DOI:** 10.1371/journal.pmed.1002175

**Published:** 2016-11-22

**Authors:** Badara Cissé, El Hadj Ba, Cheikh Sokhna, Jean Louis NDiaye, Jules F. Gomis, Yankhoba Dial, Catherine Pitt, Mouhamed NDiaye, Matthew Cairns, Ernest Faye, Magatte NDiaye, Aminata Lo, Roger Tine, Sylvain Faye, Babacar Faye, Ousmane Sy, Lansana Konate, Ekoue Kouevijdin, Clare Flach, Ousmane Faye, Jean-Francois Trape, Colin Sutherland, Fatou Ba Fall, Pape M. Thior, Oumar K. Faye, Brian Greenwood, Oumar Gaye, Paul Milligan

**Affiliations:** 1 Université Cheikh Anta Diop, Dakar, Sénégal; 2 London School of Hygiene & Tropical Medicine, London, United Kingdom; 3 Institut de Recherche pour le Développement, Dakar, Sénégal; 4 Ministère de la Santé et de la Prévention, Sénégal; Kenya Medical Research Institute - Wellcome Trust Research Programme, KENYA

## Abstract

**Background:**

Seasonal Malaria Chemoprevention (SMC) with sulfadoxine-pyrimethamine (SP) plus amodiaquine (AQ), given each month during the transmission season, is recommended for children living in areas of the Sahel where malaria transmission is highly seasonal. The recommendation for SMC is currently limited to children under five years of age, but, in many areas of seasonal transmission, the burden in older children may justify extending this age limit. This study was done to determine the effectiveness of SMC in Senegalese children up to ten years of age.

**Methods and Findings:**

SMC was introduced into three districts over three years in central Senegal using a stepped-wedge cluster-randomised design. A census of the population was undertaken and a surveillance system was established to record all deaths and to record all cases of malaria seen at health facilities. A pharmacovigilance system was put in place to detect adverse drug reactions. Fifty-four health posts were randomised. Nine started implementation of SMC in 2008, 18 in 2009, and a further 18 in 2010, with 9 remaining as controls. In the first year of implementation, SMC was delivered to children aged 3–59 months; the age range was then extended for the latter two years of the study to include children up to 10 years of age. Cluster sample surveys at the end of each transmission season were done to measure coverage of SMC and the prevalence of parasitaemia and anaemia, to monitor molecular markers of drug resistance, and to measure insecticide-treated net (ITN) use. Entomological monitoring and assessment of costs of delivery in each health post and of community attitudes to SMC were also undertaken. About 780,000 treatments were administered over three years. Coverage exceeded 80% each month. Mortality, the primary endpoint, was similar in SMC and control areas (4.6 and 4.5 per 1000 respectively in children under 5 years and 1.3 and 1.2 per 1000 in children 5-9 years of age; the overall mortality rate ratio [SMC: no SMC] was 0.90, 95% CI 0.68–1.2, *p* = 0.496). A reduction of 60% (95% CI 54%–64%, *p* < 0.001) in the incidence of malaria cases confirmed by a rapid diagnostic test (RDT) and a reduction of 69% (95% CI 65%–72%, *p* < 0.001) in the number of treatments for malaria (confirmed and unconfirmed) was observed in children. In areas where SMC was implemented, incidence of confirmed malaria in adults and in children too old to receive SMC was reduced by 26% (95% CI 18%–33%, *p* < 0.001) and the total number of treatments for malaria (confirmed and unconfirmed) in these older age groups was reduced by 29% (95% CI 21%–35%, *p* < 0.001). One hundred and twenty-three children were admitted to hospital with a diagnosis of severe malaria, with 64 in control areas and 59 in SMC areas, showing a reduction in the incidence rate of severe disease of 45% (95% CI 5%–68%, *p* = 0.031). Estimates of the reduction in the prevalence of parasitaemia at the end of the transmission season in SMC areas were 68% (95% CI 35%–85%) *p* = 0.002 in 2008, 84% (95% CI 58%–94%, *p* < 0.001) in 2009, and 30% (95% CI -130%–79%, *p* = 0.56) in 2010. SMC was well tolerated with no serious adverse reactions attributable to SMC drugs. Vomiting was the most commonly reported mild adverse event but was reported in less than 1% of treatments. The average cost of delivery was US$0.50 per child per month, but varied widely depending on the size of the health post. Limitations included the low rate of mortality, which limited our ability to detect an effect on this endpoint.

**Conclusions:**

SMC substantially reduced the incidence of outpatient cases of malaria and of severe malaria in children, but no difference in all-cause mortality was observed. Introduction of SMC was associated with an overall reduction in malaria incidence in untreated age groups. In many areas of Africa with seasonal malaria, there is a substantial burden in older children that could be prevented by SMC. SMC in older children is well tolerated and effective and can contribute to reducing malaria transmission.

**Trial Registration:**

ClinicalTrials.gov NCT00712374

## Abstract 2

In a stepped-wedge cluster-randomized trial, Paul J. Milligan and colleagues determine the effectiveness of SMC in Senegalese children up to ten years of age.

## Author Summary

**Why was this study done?:**

- SMC was originally intended for children under five years of age. In many areas of the sub-Sahel, there is a substantial burden of malaria in older children; this may justify extending the age range of SMC.
- This study was done in central Senegal to evaluate the effectiveness of SMC when given to children up to ten years of age.

**What did the researchers do and find?:**

- SMC was introduced into the area served by 54 health posts by district health teams over three years using a randomised stepped-wedge design.
- Malaria cases were detected at outpatient clinics and in hospitals, and deaths were recorded through a demographic surveillance system.
- SMC reduced the incidence of malaria in children up to ten years of age by 60%. No reduction in all-cause child deaths was observed, but the incidence of severe malaria was reduced by 45%.
- SMC administered to children up to ten years of age led to a reduction in malaria incidence in older age groups by 26%.

**What do these findings mean?:**

- SMC given to children up to ten years of age is highly effective in preventing malaria in children and can contribute to reducing overall malaria transmission.
- SMC programmes have started in 11 countries. In many of these countries, there is a substantial burden of malaria in older children. The findings of this study indicate that a much greater impact could be achieved by extending the age range of SMC.

## Introduction

In 2012, WHO recommended that children under five years of age living in areas of highly seasonal malaria transmission in the Sahel and sub-Sahel should receive seasonal malaria chemoprevention (SMC) with sulfadoxine-pyrimethamine (SP) plus amodiaquine (AQ) for up to four months of the year to prevent malaria [1]. It has been estimated that about 25 million children under five years of age resident in 15 countries live in areas suitable for SMC, defined as areas where 60% of annual cases fall in four consecutive months of the year. About 20 million of these children live in areas where estimates of the parasite rate (PfPr2-10) were consistent with an incidence of malaria in excess of 0.1 cases per child per year, areas where SMC is likely to be most cost effective [2,3]. Following regional meetings of malaria control programme managers to develop SMC implementation plans and the publication of an implementation guide detailing methods of delivery, monitoring, and evaluation of SMC [4,5], National Malaria Control Programmes have been quick to adopt this policy. SMC schemes have started in eleven countries (Burkina Faso, Cameroon, Chad, The Gambia, Ghana, Guinea, Mali, Niger, Nigeria, Senegal, and Togo). Seven of these countries are expanding access to SMC through a UNITAID-funded programme (ACCESS-SMC, [6]).

In some parts of the areas suitable for SMC, the proportional burden of malaria in older children is increasing [7], reflecting slower acquisition of natural immunity; in these areas, extending the age range for administration of SMC above five years may be justified [8]. The size of the burden in older children is often not apparent to programme managers, because standard reporting templates report the number of malaria cases in three groups (those under five years of age, those over five years of age, and pregnant women) and do not disaggregate age groups above five years of age.

This study was planned prior to the WHO policy recommendation for SMC to evaluate the effectiveness of SMC using a stepped-wedge design. The study was designed to determine the feasibility of delivering SMC on a large scale to children under five years of age, its safety, and its costs. After the first year of implementation, it was apparent that there was a substantial malaria burden in older children. The intervention was therefore expanded to include all children up to ten years of age in the second and third years of the study in order to be able to evaluate the effectiveness of SMC in this wider age group. This paper reports the study design and the results on effectiveness. The pharmacovigilance system, analysis of coverage, and the economic evaluation are reported separately [9].

## Methods

### Ethics

A series of meetings were held with local government authorities and district health staff to explain the aims and activities of the project. On the first occasion, when the intervention drugs were delivered through house-to-house visits, verbal consent to participate in the SMC programme was sought from the mother or carer of each eligible child using a standard script translated into the appropriate local language (Wolof or Serer), which mentioned the aims of the project and the potential side effects of the study drugs. Verbal consent or refusal was recorded in a register by the community worker. Consent was sought separately for participation in surveys and demographic surveillance. The study protocol was approved by the ethics committee of the London School of Hygiene & Tropical Medicine and by the Conseil Nationale de Recherche in Senegal. The same committees approved an amendment in 2009 to include older children in SMC administration. A Data Safety Monitoring Board monitored the safety aspects of the study.

### Study Site

**Fig 1:**
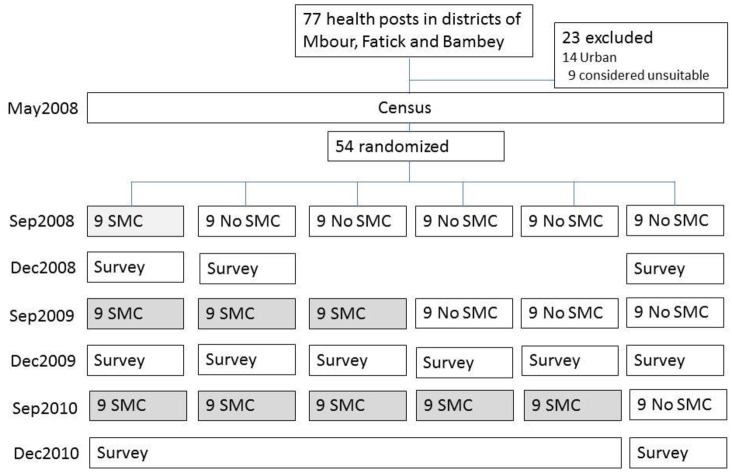
Trial profile.

**Fig 2:**
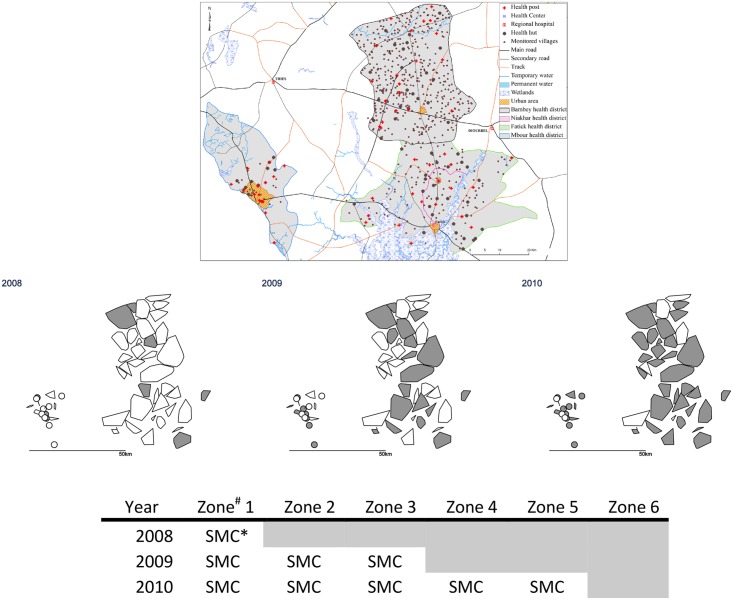
Stepped-wedge introduction of SMC in 45 health post areas over three years. The upper maps show the health district boundaries (the district of Fatick was subdivided in 2010 to create a new district of Niakhar); land cover and water bodies; and the location of villages and health facilities. In the lower maps, the polygons show the catchment areas of the 54 health posts (drawn as the convex hull of the village coordinates). ^#^ Each zone comprised nine health post areas. The nine health post areas in Zone 6 remained untreated. * In 2008, SMC was delivered to children aged 3–59 months; in other years, SMC was provided for children aged 3–119 months.

The study was undertaken in the health districts of Mbour, Bambey, and Fatick in central Senegal, which have a combined population of about 600,000 and which are served by 54 health posts and three district health centres. The trial profile is shown in Fig 1, and the study area and phased design are shown in Fig 2 and S1 Fig. (Note that Fatick district was subdivided in 2010, part of the original health district forming the new health district of Niakhar; both areas remained in the study). The climate of the study area is sudano-sahelian, with a single rainy season from July to the beginning of October; monthly mean temperature range from 24°C (December–January) to 30°C (May–June). The main vector species is *Anopheles gambiae* sensu lato (predominantly *Anopheles arabiensis)*. The mean indoor man-biting rate of *An*. *gambiae s*.*l*. ranged from 0.3 to 6 bites per night in 2008, 0.2 to 10 in 2009, and 0.2 to 22 in 2010, with similar results for outdoor biting (S1 Fig).

### Formative Research

Before the study, formative research was done to identify the most suitable methods for delivering SMC, and a pilot implementation was undertaken to assess acceptability to communities and to health staff, to measure compliance, to identify any important constraints, and to provide data to support a funding application for the main study.

### Stepped-Wedge Design

SMC (then known as intermittent preventive treatment in children [IPTc]) had been shown prior to the planning of this study to be highly effective in clinical trials [10,11], but it was questioned whether the intervention could be delivered on a large scale at reasonable cost, whether it would be acceptable to families, and whether the drugs used for SMC would be safe when deployed on a large scale. It was also unknown if SMC had an impact on mortality. These considerations guided the design and the sample size of the new study. A stepped-wedge design [12] was chosen, as it was not considered justifiable to include an individual- or community-randomised control group, and, because there was no established delivery channel, implementation needed to be phased in gradually for logistic reasons. It was considered that results from an uncontrolled “before and after” design would be difficult to interpret due to the variability in malaria incidence from year to year.

### Randomisation

Health officials and community leaders agreed to randomisation by health post but asked that intervention progressed at the same rate in all three districts. The health post was the natural cluster, as each health post is responsible for community programmes in their catchment area (median about 9,000 people) and supervises a network of community health workers. The three districts were served by 77 health posts, 54 of which were situated in rural and semi-urban areas and were included in the study. SMC was introduced in steps that were one year apart—9 health posts implemented in year 1, an additional 18 in year 2, and an additional 18 in year 3, leaving 9 that would start in year 4 if the study was extended (Fig 1, S1 Table, and S2 Fig). Constrained randomisation was used to avoid chance imbalance between intervention and control areas at each step. Data on the key outcomes (confirmed malaria incidence, all-cause deaths) by cluster were not available prior to the start of the trial, so the randomisation had to be based on proxy variables (distance from river, distance from health centre, population size, number of villages). An assessment of the performance and experience of the staff at each health post (on a 3-point scale) was also included to avoid imbalance in ability to implement SMC. A geographical constraint was included to avoid randomisations that resulted in clumps of intervention and control clusters. The randomisation process was kept secret; health staff knew their status for that year (implement or control) only at the start of the year. Further details are provided in S3 Text.

### Delivery

Delivery was organised by health post using community health workers who visited each household once per month during the transmission seasons of 2008, 2009, and 2010 (in mid-September, mid-October, and mid-November) to administer SMC to children aged 3–59 months (in 2008) and to children up to 10 years of age in 2009 and 2010. The dose of SP and the first dose of AQ was administered by the health worker or by the mother/carer under supervision by the health worker; the remaining daily doses of AQ were left with the mother to administer at home over the next two days. Dates of SMC administration were documented by the health worker in registers and on a family-held record card. Usage and wastage of drugs was tallied. Coverage was estimated independently from surveys each year, sampling from each intervention cluster to yield an overall estimate of annual coverage.

### Drugs

In 2008, co-blister packs of Dualkin (Pfizer) (SP [500/25 mg tablets] and AQ [200 mg tablets]) were used. These tablets had to be broken and crushed to make half doses for infants. In 2009, 200-mg AQ tablets (Chongqing Qinyang Pharmaceutical) and 500/25-mg SP (Shijizhuang Ouyi Pharmaceutical) tablets were used, and, in 2010, sweetened dispersible tablets of SP (500/25 mg) and of AQ (153 mg) (Kinapharma, Ghana) were given. Clinical episodes of malaria were treated with AQ-artesunate or quinine in 2008 and with artemether-lumefantrine or quinine in 2009 and 2010, following a clinical algorithm that required suspected cases to be tested with an RDT (Paracheck-Pf test, Orchid Biomedical Systems, Goa, India). Amoxicillin and cotrimoxazole were the most commonly used antibiotics.

### Surveillance

Parasitological diagnosis for malaria was introduced nationally by the Senegalese Ministry of Health the same year our project started. Malaria cases were detected at health facilities by a government nurse or physician at hospitals, district health centres, and health posts, by nurses at mission clinics, or by a community health worker working in “health huts” and in the community. The percentage of malaria treatments that were based on confirmation by RDT or slide was 69% in 2008, 97% in 2009, and 96% in 2010. The number of health huts in the study area increased from 47 in 2008 to 76 in 2010. The breakdown by study zone is shown in S3 Text. Apart from this expansion, which occurred in all zones of the study area, there were no major changes in the level of surveillance. Training workshops were held for health post nurses, and close contact was maintained with them through supervisors who visited each health facility once every one to three months, to ensure guidelines were followed and to promote good record-keeping. Confirmation of a clinical diagnosis of malaria was made using an RDT, following an algorithm that required measuring axillary temperature with a thermometer and testing with an RDT (First Response) all persons with fever (temperature ≥37.5°C) who did not have signs of respiratory infection or other obvious causes of the fever. Malaria cases were linked to clusters based on village of residence (we did not attempt to link cases to household members listed in the Demographic Surveillance System [DSS]). Surveillance was maintained in all age groups to allow estimation of the indirect effect of the intervention in terms of a reduction in incidence of malaria in older age groups.

A pharmacovigilance system was established based on the national system but strengthened through training, supervision, and use of SMS messaging. A simplified DSS was set up, with household visits undertaken approximately every ten months. Deaths were recorded through DSS rounds (referred to as DSS1, conducted from October 2008 to June 2009, DSS2 conducted from October 2009 to July 2010, and DSS3 from October 2010 to December 2010). In 13 health posts, village reporters were trained to record any deaths in their communities, and verbal autopsies were undertaken in these cases. Cluster sample surveys of children were conducted at the end of each transmission season to measure SMC coverage and to take a finger-prick blood sample for parasitology and measurement of haemoglobin (Hb) concentration. Households were randomly selected from the DSS, and all children who would have been eligible for SMC resident in those households were included in the survey. Surveillance for malaria and for mortality was continued in 2011 to look for rebound effects.

A series of in-depth interviews were held with mothers and carers, community leaders, community health workers, health post nurses, and district medical staff using structured interview guides to find out their views on SMC, their knowledge of its benefits and risks, and the acceptability of the intervention. Interviews were recorded and transcribed into French, and transcripts were analysed.

### Laboratory Methods

A finger-prick blood sample was taken during the cross-sectional surveys to make thick films, to take blood spots on filter paper for molecular analysis, and for measurement of Hb concentration using a portable Haemocue machine (Haemocue AB, Ängelholm, Sweden). Slides were dried at room temperature for 48 hours and stained with 6% Giemsa solution. Slide reading was done at the malaria laboratory of the Institut de Recherche pour le Développement (IRD). All positive slides and a sample of negative slide were re-read by a second reader; if readings disagreed (about slide positivity or if there was a more than 2-fold difference in parasite density), a third reading was done, and the geometric mean density of the closest readings were used if the slide was judged positive. A subset of slides were read at the Parasitology Department of the Université Cheikh Anta Diop (UCAD) of Dakar for quality control purposes. Further details are given in S3 Text. Two hundred high-power fields were read to determine parasitaemia and presence of other species (*Plasmodium malariae*, *Plasmodium ovale*, and *Borrelia*) before a slide was declared negative. *Plasmodium falciparum* parasitemia density was calculated as the number of parasites per 100 leukocytes, expressed per μl assuming 8,000 white blood cells per microliter of blood. Gametocyte densities were estimated based on the number of gametocytes seen in 200 microscopic fields and the average number of white blood cells seen per field. Samples found positive for *P*. *falciparum* by microscopy were analysed by reverse transcription PCR (RT-PCR) and sequencing for the presence of mutations in the pfdhfr, pfdhps, pfmdr1, and pfcrt genes associated with resistance to SMC drugs. Children with an Hb < 7 g/dL were referred to the health post, where the child was given a course of iron tablets; severely anaemic children were referred to the district health centre for blood transfusion if required. Children with a fever were referred to the health post, their slide was read promptly, and the results were provided to the clinic. Samples of the tablets used were tested at the Laboratoire National de Contrôle des Médicaments, Dakar for drug content and dissolution.

### Statistical Methods

The primary trial outcomes were all-cause mortality, the incidence of RDT-confirmed malaria treated as outpatients, and the incidence of known drug-related adverse events. Secondary outcomes were coverage of SMC, the prevalence of parasitaemia and anaemia at the end of each transmission season, the prevalence of molecular markers associated with resistance to SP and to AQ, and the incidence of severe malaria defined as admission to hospital for at least 24 hours with a primary diagnosis of malaria. Sample size calculations to detect an effect on all-causes mortality were done for a range of scenarios, with the assumption that the mortality rate (all causes) would be 20 per 1,000 in children 3–59 months of age (based on estimates from the Niakhar DSS) and assuming 30% of deaths were due to malaria (determined from verbal autopsies in Niakhar and the proportion of deaths in hospital attributed to malaria). We also assumed a coefficient of variation between health posts of 0.25 to 0.3 and SMC effectiveness against malaria to be 70%. Calculations using the method of Hemming and Girling [13] for the stepped-wedge design, assuming k = 0.3 and a median population of children aged 3–59 months of 1,400 per cluster, gave a power of about 17%, 52%, and 99% for reductions of 5%, 10%, and 20%, respectively, if the mortality rate was 10/1,000 and 11%, 32%, and 86% power for the same reductions if the rate was 20/1,000.

Effectiveness of SMC was estimated by fitting a Poisson regression model to the data on the number of deaths and the number of RDT-confirmed malaria cases in each health post occurring in the period starting from the date of the first round of SMC and ending one month after the last round of SMC each year. The number of person-months at risk obtained from the DSS was included as an offset, and a gamma-distributed random effect was used to allow for correlation within clusters. Covariates included in the model were age group, calendar year, and indicator variables for the effect of SMC in children (under five years in 2008 and under ten years in 2009 and 2010) and for the indirect effect of SMC in older age groups. Interaction terms were included to compare effects in the two age groups, and combined effects were estimated where there was no evidence of interaction. The indicator variable representing SMC direct effects was set to 1 if that age group received SMC in that cluster in that year and set to 0 otherwise. The indicator variable for indirect effects was set to 1 for all non-SMC age groups if SMC was implemented in children in that cluster and set to zero otherwise. Thus the direct effect that was estimated represents the sum of direct and indirect effects, and the indirect effect estimated represents the indirect effect only. Rebound effects were assessed by comparing the incidence of malaria during 2011 and 2012, after intervention had stopped, in children living in areas where SMC had been provided for up to three years, with children of the same age in areas that did not have SMC.

This study is registered on www.clinicaltrials.gov, number NCT 00712374.

## Results

A census undertaken in May 2008 identified approximately 600,000 persons who were normally resident in the study area, living in 1,247 villages. Coverage with long-lasting insecticide treated nets (LLIN) in children was similar in all zones, ranging from 45% to 59% in the first year of the study and increasing to 67% to 77% by the third year (Table 1). SMC implementation started in September 2008 in Zone 1, treating about 14,000 children aged 3–59 months each month (mid-September, mid-October, and mid-November). About 90,000 children under ten years of age were treated in 2009, and about 160,000 were treated in 2010.

**Table 1:** Characteristics of the randomised intervention zones.

Variable	Zone 1	Zone 2	Zone 3	Zone 4	Zone 5	Zone 6
Number of clusters (postes de santé)	9	9	9	9	9	9
Year SMC started	2008	2009	2009	2010	2010	control
Number of villages	208	221	221	240	191	166
Population <10yrs (Sep 2009)	38,538	37,011	30,080	34,147	34,470	23,064
Total population (Sep 2009)	127,248	108,884	101,929	104,672	109,147	74,132
LLIN coverage (all ages)^#^ DSS1 (Oct 2008–Jun 2009)	40%	41%	45%	52%	44%	49%
DSS2 (Oct 2009–Jul 2010)	61%	62%	66%	70%	56%	60%
DSS3 (Oct 2010–Dec 2010)	65%	67%	70%	71%	65%	63%
LLIN coverage^##^ (children <10yrs) DSS1(Oct 2008–Jun 2009)	46%	45%	55%	59%	49%	58%
DSS2(Oct 2009–Jul 2010)	70%	69%	72%	78%	65%	69%
DSS3(Oct 2010–Dec 2010)	67%	71%	75%	77%	71%	69%
Mean distance to poste de santé, km:						
Unweighted*	3.9	4.5	3.6	3.1	2.7	4.5
Weighted by population	2.1	3.7	2.9	2.4	2.2	3.0
Mean distance to district health centre, km:						
Unweighted	21	11	13	17	9.5	22
Weighted by population	12	9	11	15	8.4	17
Median household size	9	10	9	10	9	9
% households in the poorest quintile group**	12%	30%	22%	20%	19%	16%
Characteristics of heads of households:						
% with at least primary education	28%	16%	33%	16%	25%	29%
Religion (%muslim)	95%	95%	82%	97%	88%	91%
Ethnicity:						
Wolof	43%	19%	34%	22%	33%	38%
Serer	33%	75%	50%	74%	51%	44%
Pulaar	15%	3.4%	6.9%	1.9%	9.7%	10%
Other	9.8%	1.9%	8.8%	1.6%	6.0%	7.9%

^#^No. surveyed 422,164 (DSS1), 464,642 (DSS2), and 90,276 (DSS3). LLIN use was defined as an LLIN present on the bed where the person usually sleeps.

^##^No. surveyed 149,556 (DSS1), 139,011 (DSS2), and 28,006 (DSS3).

*Mean distance from the village to the health post. Unweighted distances relate to ease of intervention delivery; weighted distances reflect population access to health care.

**The proportion of households in the poorest fifth of all households in the study area based on household assets; assessed in DSS2.

### Coverage and Adherence

The proportion of children who received three monthly SMC treatments in 2008 was 93% (95% CI 91%–95%), estimated from a survey of 1,019 children in 9 health post areas; the corresponding figure in 2009 was 84% (95% CI 82%–87%) (*n* = 3,397) and 93% in 2010 (95% CI 91%–96%) (*n* = 882). A high level of adherence to daily doses was reported. The main reason for missed doses was that the caregiver was away when the health worker visited. Fewer than 1% refused, and less than 1% of children missed SMC because of illness on the day of treatment; those who did miss SMC due to illness on the day of treatment were referred to the health facility. When asked about adherence to the two doses of AQ left for the caregiver to administer, 9.6% reported difficulties giving these doses.

### Mortality

During the transmission seasons, the mortality rate among children aged 3–59 months in SMC areas was 4.6 per 1,000 child-years at risk (197 deaths) and 4.5/1,000 in control areas (159 deaths). Among children aged 5–9 years, the mortality rates were 1.30/1,000 in SMC areas (45 deaths) and 1.2/1,000 in control areas (43 deaths). Mortality in infants under three months of age was 19/1,000. The mortality rate ratio SMC: control given by a random effect model that included cluster, time, and age effects was 0.89 (95% CI 0.65–1.2, *p* = 0.442) for the 3–59 months age group and 0.97 (95% CI 0.61–1.6, *p* = 0.916) for the 5- to 9-year-olds. The pooled estimate for both age groups combined was 0.90 (95% CI 0.68–1.2 *p* = 0.496) (S3 Fig, S2 Table).

### Outpatient Cases of Malaria

**Fig 3:**
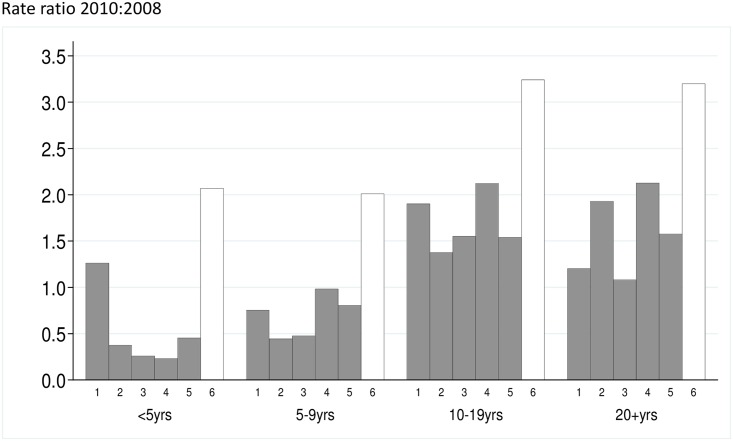
The malaria incidence rate ratio after to before introduction of SMC is shown for five study zones (1–5) where SMC was introduced, and the control area (Zone 6) that remained without SMC. The *y*-axis is the rate ratio comparing the years 2010:2008. Rate ratios for each age group are shown.

A total of 1,001 RDT-confirmed cases of malaria were recorded in children <10 years in 2008, 310 in 2009, and 1,111 in 2010. The number treated for malaria without an RDT being performed was 1,231 in 2008 but fell to 73 in 2009 and 17 in 2010, following expansion of a new national policy of “test and treat” in 2009. In Zone 6, which did not receive SMC, the incidence of RDT-confirmed malaria during the transmission season was 2.0/1,000 in children under five years of age in 2008, 0.2/1,000 in 2009, and 4.3/1,000 in 2010 (S4 and S5 Figs). The reasons for the very low incidence in 2009 are not known. Rates of confirmed cases in under 5′s in SMC areas were 2.8,0.36 and 2.0 per1000 in the 2008, 2009 and 2010 transmission seasons respectively and 2.2, 0.51 and 4.3 in control areas in the same years. In the 5-9 age group the rates were 0.83 and 4.1 per 1000 in SMC areas in 2009 and 2010 respectively and 1.5 and 10.0 in control areas in the same years (S3 Table). The incidence rate ratio comparing SMC and control areas, adjusted for effects of calendar time, for RDT-confirmed malaria during the transmission season was 0.43 In children under five years of age and 0.39 for children five to nine years of age (Table 2), giving reductions of 57% (95% CI 48%–63%) in the younger group and 61% (95% CI 55%–67%) in the older group, an overall effectiveness of 60% (95% CI 54%–64%, *p* < 0.001). The reduction in all cases treated for malaria (confirmed and unconfirmed) was 69% (95% CI 65%–72%, *p* < 0.001). Among age groups too old to receive SMC, incidence was reduced by 26% (95% CI 18%–33%, *p* < 0.001) (RDT-confirmed), or 29% (95% CI 21%–35%, *p* < 0.001) (confirmed and unconfirmed cases) in areas where SMC was delivered to children compared to control areas (Fig 3). When cases of malaria throughout the year were included in the analysis, the reduction in confirmed cases in children under ten years of age was 48% (95% CI 42%–54%, *p* < 0.001) and in total malaria treatments was 60% (95% CI 56%–63%). The indirect effect in older age groups was an overall reduction of 20% (95% CI 12%–27%) in confirmed cases and 20% (95% CI 13%–25%) in total treatments (S3 Table).

**Table 2:** Rate ratios for the effect of SMC on the incidence of malaria. Rate ratios comparing SMC and non-SMC areas are shown for children and for older age groups. Effects of age and calendar year are also shown. Analyses were repeated using only RDT-confirmed malaria cases and cases treated for malaria with or without confirmation.

		Incidence rate ratio (95% CI)	
Variable		All malaria cases	RDT-confirmed
Direct effect of SMC^1^			
	No SMC	1	1
	SMC	0.31 (0.28–0.35)	0.40 (0.36–0.46)
Indirect effect of SMC^2^			
	No SMC in the area	1	1
	SMC in the area	0.71 (0.65–0.79)	0.74 (0.67–0.82)
Age	<5 yrs	1	1
	5–9 yrs	1.58 (1.45–1.72)	2.01 (1.82–2.22)
	10–19 yrs	1.68 (1.54–1.83)	2.40 (2.17–2.65)
	20–29 yrs	1.46 (1.34–1.60)	2.08 (1.87–2.31)
	30–49 yrs	1.18 (1.07–1.29)	1.63 (1.46–1.81)
	50+ yrs	0.73 (0.66–0.82)	1.03 (0.90–1.17)
Year	2008	1	1
	2009	0.38 (0.35–0.41)	0.51 (0.47–0.56)
	2010	1.98 (1.80–2.17)	2.51 (2.28–2.77)

^1^ Direct effects in SMC age groups.

^2^ Indirect effects (adults and children too old to receive SMC, living in areas where SMC was delivered). *p*-Values for the SMC effects were all <0.001.

Note: The variance of the gamma-distributed random effect was 0.43, giving an underlying coefficient of variation of the incidence rate per cluster of 0.66.

### Severe Malaria

One hundred and twenty-three children were admitted to hospital with a diagnosis of severe malaria, with 50 under 5 years of age (25 in SMC areas and 25 in control areas, rates per 100 child-years of 0.48 and 0.53, respectively) and 73 children 5–9 years of age (22 before SMC started in this age group, 34 in SMC areas, and 17 in control areas, rates of 0.95 and 1.2, respectively), giving a combined rate ratio of 0.55 and an overall reduction of 45% (95% CI 5%–68%, *p* = 0.031), with no evidence of difference between the age groups (interaction *p*-value 0.224) (S4 Table).

### Prevalence of Parasitaemia and Anaemia at the End of the Transmission Season

SMC was associated with a reduction in the prevalence of *P*. *falciparum* parasitaemia in both age groups. In 2008, in children under five years of age, the prevalence was 5.1% in control areas and 1.6% in SMC areas, a reduction of 68% (95% CI 35%–85%) *p* = 0.002. In children under ten years of age, in 2009, the prevalence was 1.3% and 0.22% in control and SMC areas (reduction of 84% (95% CI 58%–94%, *p* < 0.01), and in 2010, 2.5% and 1.86, respectively, a reduction of 30% (95% CI -130%–79%, *p* = 0.56) (Table 3). Gametocyte carriage was reduced in SMC areas in 2008 (2.6% compared to 0.64%, prevalence ratio 0.28 [95% CI 0.10–0.75]). In 2009 and 2010, the number of gametocyte carriers was too low to measure any difference between SMC and control areas (S2 Table). Mean Hb concentration, which was measured in 2008 and 2009, was similar in SMC and non-SMC areas in each year (S5 Table). The prevalence of moderate anaemia in children under five years of age was 27% in SMC areas and 29% in non-SMC areas in 2008, a prevalence ratio of 0.92 (95% CI 0.79–1.07), and 27.9% and 27.6% in 2009, a prevalence ratio of 1.0 (95% CI 0.86–1.15). The prevalence of severe anaemia (Hb < 6 g/dL) was 1.07% and 0.32%, prevalence ratio 0.30 (95% CI 0.07–1.19) in 2008 and 1.0% and 2.2%, prevalence ratio 2.1 (95% CI 0.93–4.9) in 2009. In children 5–9 years of age in 2009, the prevalence of moderate anaemia was 8.9% and 9.2%, prevalence ratio 1.0 (95% CI 0.86–1.15), and the prevalence of severe anaemia was 1.21% and 1.20%, prevalence ratio 1.01 (95% CI 0.44–2.33) (S6 and S7 Tables).

**Table 3:** Prevalence of *P*. *falciparum* parasitaemia in children under five years old (2008) and under ten years old (2009 and 2010)* in SMC and control areas at the end of the malaria transmission season.

		Prevalence(95% CI)	Prevalence ratio (95% CI)	*p*-value
2008	Non-SMC area	5.1% (2.3%–7.9%)	1	
	SMC area	1.6% (0.86%–2.4%)	0.32 (0.15–0.65)	0.002
2009	Non-SMC area	1.3% (0.58%–2.1%)	1	
	SMC area	0.22% (0.04%–0.39%)	0.16 (0.06–0.42)	<0.001
2010	Non-SMC area	2.5% (0.27%–4.7%)	1	
	SMC area	1.8% (0.43%–3.1%)	0.70 (0.21–2.3)	0.56

* In 2008, children who were aged 3–59 months in September of that year; in 2009 and in 2010, children who were aged 3–119 months in September of that year.

### Markers of Resistance to SMC Drugs

One hundred and sixty-one samples positive for *P*. *falciparum* parasitaemia were typed, 48 from SMC areas and 113 from non-SMC areas. The proportion with parasites with *pfdhfr* and *pfdhps* mutations was similar in SMC and non-SMC areas (prevalence ratios SMC: non-SMC, adjusted for study year, were 0.90 [95% CI 0.79–1.02] for the *pfdhfr* triple mutation and 0.98 [95% CI 0.80–1.21] for the *pfdhps* mutation at codon 437), whereas the CVIET haplotypes of *pfcrt* and the *86Y* polymorphism of pfmdr1 were more common in SMC areas (prevalence ratios 1.46 [95% CI 0.70–3.05] for *pfcrt* CVIET, 1.86 [95% CI 1.04–3.34] for *pfmdr1* 86Y, and 1.09 [95% CI 0.82–1.47] for *pfmr1* 184F). When the overall prevalence of pfdhfr, pfdhps, pfcrt, and pfmdr1 mutations was compared (including both infected and uninfected children in the denominator), the prevalences were lower in SMC areas than in control areas [14].

### Adverse Events

No cases of Stevens–Johnson syndrome were seen in children who received SMC. One child had an extra-pyramidal syndrome associated with AQ taken during SMC but recovered. Three serious adverse events were considered possibly related to SMC: an acute diarrhoeal illness, a case of rash and facial oedema, and a case of jaundice. The average percentage of children who attended clinic within ten days of an SMC round with symptoms attributed to SMC drugs was 0.1% in 2008, 0.2% in 2009, and 0.14% in 2010. The most commonly reported symptom was vomiting. Details of adverse events are described in [9].

### Costs

The average financial cost of delivery was US$0.50 per child per month, and economic cost was US$0.59. Estimates for each health post varied widely depending on the size of the health post.

### Incidence of Malaria in SMC and Control Areas in the Year after Intervention

Children could have received SMC for up to three years, depending on their age and implementation zone. The number of previous years of SMC can be defined for each age group in each zone (S8 Table); for example, those living in areas where SMC implementation started in 2008, if they were aged 3–59 months when SMC started in 2008, could have received SMC for up to three years; these children would be aged from three years and three months to seven years at the start of the 2011 transmission season. When the incidence of malaria in 2011 was compared in relation to the number of years of SMC, the rate ratio for three years of previous SMC, compared with no previous SMC, was 1.18 (95% CI 0.84–1.67); for two years of SMC, 1.09 (95% CI 0.82–1.45); and for one year of SMC, 0.77 (95% CI 0.59–1.01), after adjusting for effects of age.

## Discussion

This trial showed that SMC delivered to children under ten years of age by district health teams on a large scale was highly effective in reducing the malaria burden in young and older children by at least 60%, that the intervention was well tolerated and well accepted by communities, and that high coverage could be achieved at moderate cost. There was a substantial reduction in the incidence of malaria and in the prevalence of parasitaemia, and, importantly, our results were consistent with an indirect benefit in age groups too old to receive SMC themselves.

We found a reduction in the incidence of severe malaria, but we did not observe a reduction in all-cause mortality. No reduction in the prevalence of anaemia was observed. In previous studies [14], the effect of SMC on anaemia has differed between studies; this may reflect differences in the extent to which malaria is an important cause of anaemia in different populations.

In areas of intense transmission, the main burden of severe malaria disease is in young children, but, as the intensity of transmission decreases, an increasing proportion of cases occur at older ages, reflecting slower acquisition of natural immunity; in these areas, wider age group could be included in SMC programmes.

Despite high coverage and the contribution of a herd effect, SMC effectiveness was somewhat lower than the efficacy observed in clinical trials (efficacy of 75% against uncomplicated and severe malaria, [15]). This may be partly due to the use of RDTs to diagnose malaria. These can give a false positive result, as the HRP (histidine-rich protein) persists after infections have been treated, reducing specificity and biasing effectiveness estimates downwards. Care was taken to assign malaria cases to clusters on the basis of their place of residence, but there could have been some misclassification, which would also reduce estimates of effectiveness. We may have somewhat overestimated coverage by sampling, at the end of the transmission season, the population resident at that time, excluding families that were present for only parts of the transmission season whose children may have missed SMC doses. Our results could have been influenced by effects of other interventions: these included distribution of LLINs and a campaign of mass treatment with azithromycin in Bambey district. However, randomisation, which was balanced by district, should have minimised the impact of this source of bias. During one year of the trial, malaria incidence was unexpectedly very low. The reasons for this are not known, as malaria surveillance methods were unchanged and no reduction in mosquito density was seen in that year in the four areas where entomological monitoring was done.

An effect on transmission brought about by the clearance of infection in a substantial proportion of the population (children under ten years of age represent about 30% of the population and account for about 40% of all outpatient malaria cases) is not unexpected. The use of microscopy to detect gametocytes may have underestimated the prevalence of gametocyte carriage, but there was a substantial reduction in the prevalence of asexual parasitaemia, and it is likely this was accompanied by a reduction in gametocyte prevalence. We were not able to measure an effect of SMC on entomological inoculation rates; this would be challenging due to the very low sporozoite rates. The randomisation zones were well balanced at baseline, and the stepped-wedge design permits adjustment for pre-intervention incidence, so a chance imbalance is unlikely to account for the observed findings.

We found no evidence that children who had received SMC for up to three years were at increased risk of malaria the year after SMC stopped. This is consistent with the relatively low incidence of malaria and the consequently slow pace of naturally acquired immunity in untreated children in the study area.

SMC programmes will increase selection pressure for drug resistance, but the rate at which this will occur is not known. In our study, children who had *P*. *falciparum* infection at the end of the transmission season were more likely to carry the *pfmdr1* 86Y mutation associated with AQ resistance if they came from areas where SMC had been implemented [14], but the effect of this on the spread of resistance may be mitigated to some extent by the much lower prevalence of infection in SMC areas; the overall proportion of children carrying parasites with these mutations was consequently lower in SMC areas than in areas where SMC had not been implemented.

We did not observe an effect on all-cause mortality. There had been a gradual reduction in mortality rates in the years before the study related to improved vaccination coverage and access to primary health care [16], but the sudden drop in mortality in our study was unexpected. It may partly reflect a reduction in malaria transmission associated with scaling up of universal coverage of LLINs and may also be related to increased use of antibiotics. When RDTs for malaria were introduced in Senegal in 2007 and 2008, there was increased use of antibiotics, as guidelines for health facility workers stipulated that persons who tested negative for malaria should be given a broad-spectrum antibiotic. Although we were not able to demonstrate an effect on all-cause mortality, a reduction in the incidence of severe malaria was observed; there may, therefore, have been a reduction in malaria-specific mortality, but the overall number of deaths and the proportion due to malaria was too low for this effect to be measured.

Including older children increases the cost of SMC programmes, but in our study, which employed door-to-door delivery, did not greatly increase the overall time required for delivery, as older children could readily be treated during health worker visits.

A Technical Expert Group of WHO reviewed evidence about SMC in 2011 [2], including preliminary results from this study. SMC was recommended for high-incidence areas [1]; our study area fell outside these recommendations on the basis of a low incidence of malaria, and so our implementation was not expanded, but monitoring was continued to assess rebound effects. SMC is now being implemented in the south of Senegal, where the burden is higher, for children up to ten years of age. In 2015, SMC programmes took place in nine countries, in about 7 million children. Apart from in Senegal, SMC is currently being provided only for children up to five years of age, but, in many areas, the burden in older children may justify extending this range. Our study shows that, in some areas, expanding the age range for SMC could have a substantial impact on the malaria burden and could contribute to reducing malaria transmission.

## Supporting Information

S1 Fig Map of study area and biting rates from human landing catches in the four monitoring sites. (DOCX)

S2 Fig Study design. (DOCX)

S3 Fig Mortality in children in treated and untreated zones. (DOCX)

S4 Fig Malaria incidence in children in treated and untreated zones. (DOCX)

S5 Fig Seasonal pattern of malaria. The number of cases (RDT confirmed) per month in each zone, by age group.
(DOCX)

S1 Table Stepped-wedge design layout. (DOCX)

S2 Table Mortality rate ratios during the transmission season of each year, by age group. (DOCX)

S3 Table Effect of SMC in each age group during the transmission season (from September 15 to December 15 each year) and when cases throughout the year were included. (DOCX)

S4 Table Incidence of severe malaria. (DOCX)

S5 Table Prevalence of gametocyte carriage in children under five years old (2008) and under ten years old (2009 and 2010) in SMC and control areas (in 2008, children who were aged 3–59 months in September of that year; in 2009, and in 2010, children who were aged 3–119 months in September of that year). (DOCX)

S6 Table Mean Hb concentration at the end of the 2008 and 2009 transmission seasons in SMC and non-SMC areas. (DOCX)

S7 Table The prevalence of moderate (Hb < 11 g/dL) and severe (Hb < 6 g/dL) anaemia at the end of the 2008 and 2009 transmission seasons in SMC and non-SMC areas. (DOCX)

S8 Table Rebound effects: age-adjusted incidence rate ratios (confirmed malaria in the 2011 transmission season) in relation to the number of previous years of SMC. (DOCX)

S1 Text Protocol. (DOC)

S2 Text CONSORT checklist. (DOCX)

S3 Text Methods. (DOCX)

## Abbreviations

AQ: amodiaquine
HRP: histidine-rich protein
ITN: insecticide-treated nets
LLIN: long-lasting insecticide-treated nets
SMC: seasonal malaria chemoprevention
SP: sulfadoxine-pyrimethamine
