# Supplementary material for: Effectiveness of Seasonal Malaria Chemoprevention in Children under Ten Years of Age in Senegal: A Stepped-Wedge Cluster-Randomised Trial
Source: PLoS Med. 2016 Nov 22;13(11):e1002175. doi: 10.1371/journal.pmed.1002175 (PMC5119693; doi:10.1371/journal.pmed.1002175)

S1 Fig


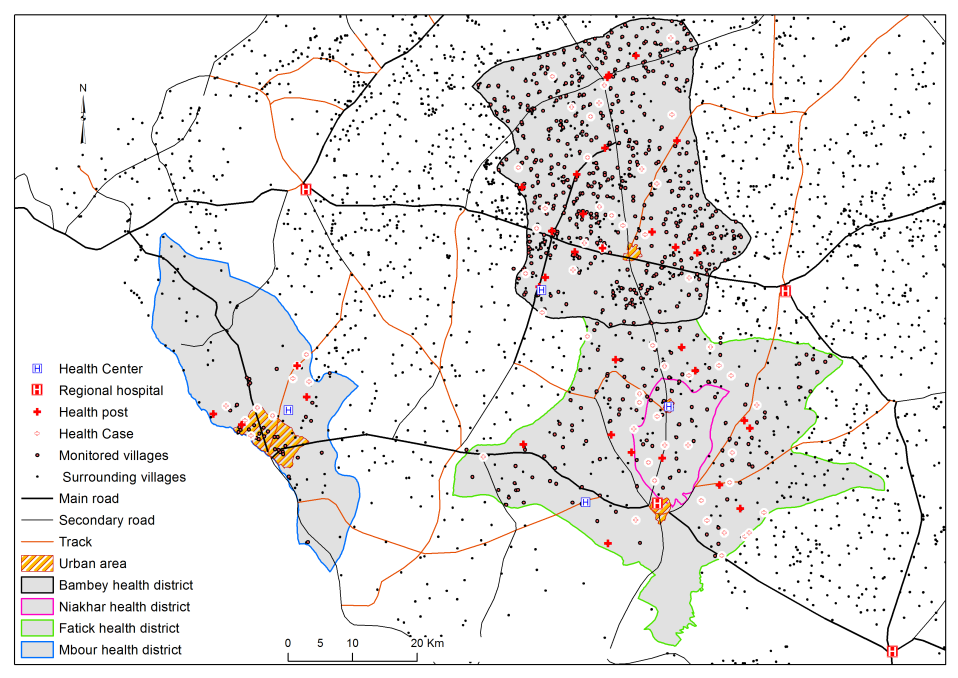
Map of the study area showing surrounding villages


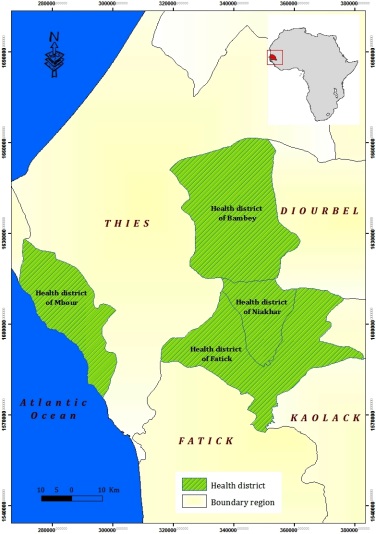


Biting rates from human landing catches in the four monitoring sites:


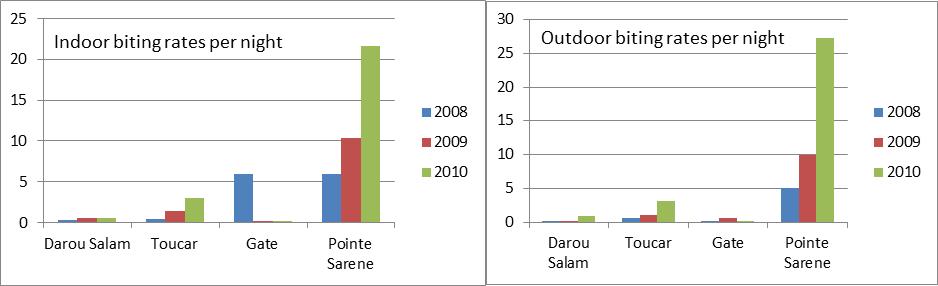

Supplement: S1 Fig — (DOCX) [file pmed.1002175.s001.docx]
