# Supplementary material for: Effectiveness of Seasonal Malaria Chemoprevention in Children under Ten Years of Age in Senegal: A Stepped-Wedge Cluster-Randomised Trial
Source: PLoS Med. 2016 Nov 22;13(11):e1002175. doi: 10.1371/journal.pmed.1002175 (PMC5119693; doi:10.1371/journal.pmed.1002175)
Supplement: S4 Fig — (DOCX) [file pmed.1002175.s004.docx]

S4 Fig Malaria incidence in children in treated and untreated zones*.

*Gray bars indicate incidence in areas where SMC was implemented. Malaria treatments (confirmed and unconfirmed) during the transmission season.
