# Supplementary material for: Effectiveness of Seasonal Malaria Chemoprevention in Children under Ten Years of Age in Senegal: A Stepped-Wedge Cluster-Randomised Trial
Source: PLoS Med. 2016 Nov 22;13(11):e1002175. doi: 10.1371/journal.pmed.1002175 (PMC5119693; doi:10.1371/journal.pmed.1002175)
Supplement: S5 Fig — The number of cases (RDT confirmed) per month in each zone, by age group. (DOCX) [file pmed.1002175.s005.docx]

S5 Fig Seasonal pattern of malaria. The number of cases (RDT confirmed) per month in each zone, by age group:
