# Supplementary material for: Effectiveness of Seasonal Malaria Chemoprevention in Children under Ten Years of Age in Senegal: A Stepped-Wedge Cluster-Randomised Trial
Source: PLoS Med. 2016 Nov 22;13(11):e1002175. doi: 10.1371/journal.pmed.1002175 (PMC5119693; doi:10.1371/journal.pmed.1002175)
Supplement: S2 Table — (DOCX) [file pmed.1002175.s007.docx]

S2 Table Mortality rate ratios. Poisson regression (Stata version 12 , College Station, Texas) was used, relating the number of deaths of all causes to the person time at risk during the three months of the intervention period each year, starting from the date of the first round of SMC and ending one month after the last round of SMC, with year, age band and zone as covariates, and a gamma-distributed random effect to allow for correlation within health posts, with an indicator variable for the age group and cluster to indicate whether SMC was delivered.

| Variable | Categories | Mortality rate ratio (95%CI) | P-value |
| --- | --- | --- | --- |
| SMC in children | No SMC | 1 |  |
| 3-59months | SMC | 0.89 (0.65,1.20) | 0.442 |
| SMC in children | No SMC | 1 |  |
| 60-119months | SMC | 0.97 (0.61,1.57) | 0.916 |
| Year | 2008 | 1 |  |
|  | 2009 | 0.63 (0.49,0.81) | 0.000 |
|  | 2010 | 1.07 (0.82,1.39) | 0.631 |
| Age group | <3months | 1 |  |
|  | 3-59months | 0.25 (0.19,0.34) | 0.000 |
|  | 60-119months | 0.066 (0.046,0.097) | 0.000 |
| Zone | 1 | 1 |  |
|  | 2 | 1.10 (0.67,1.79) | 0.708 |
|  | 3 | 0.71 (0.43,1.18) | 0.190 |
|  | 4 | 0.92 (0.55,1.53) | 0.746 |
|  | 5 | 0.79 (0.48,1.31) | 0.362 |
|  | 6 | 0.77 (0.44,1.36) | 0.373 |

When data for the whole year were included (August to December for 2008 and January to December in 2009 and 2010), the adjusted mortality rate ratios for SMC were 0.98 (95%CI 0.82,1.2) P=0.869 in the younger age group and 0.91 (95%CI 0.69,1.2) P=0.523 in children 5-9years.

Mortality (No of deaths/1000s of Person years) in each age group, per year (from 1^st^ August in 2008, from Jan to Dec in other years). Shaded cells correspond to zones and age groups that received SMC.

|  | <3months | | | |  | 3-59months | | | |  | 5-9years | | | |
| --- | --- | --- | --- | --- | --- | --- | --- | --- | --- | --- | --- | --- | --- | --- |
| Zone: | 2008 | 2009 | 2010 | 2011 |  | 2008 | 2009 | 2010 | 2011 |  | 2008 | 2009 | 2010 | 2011 |
| 1 | 15  (0.5192) | 15  (1.1490) | 26  (1.0061) | 14  (0.7230) |  | 49  (8.3696) | 66  (20.4822) | 94  (20.9939) | 63  (20.6719) |  | 9  (7.2964) | 13  (18.3429) | 17  (19.5146) | 11  (20.5795) |
| 2 | 9  (0.4707) | 10  (1.1670) | 20  (1.0674) | 15  (0.8253) |  | 59  (8.0490) | 115  (19.6020) | 121  (20.0777) | 73  (20.0680) |  | 15  (6.7992) | 19  (17.2961) | 14  (18.3371) | 11  (18.9074) |
| 3 | 8  (0.3793) | 16  (0.9055) | 21  (0.8962) | 11  (0.6073) |  | 25  (6.4815) | 44  (15.7035) | 63  (16.2292) | 45  (16.7309) |  | 6  (5.8262) | 24  (14.4528) | 19  (14.8957) | 16  (15.8473) |
| 4 | 4  (0.4491) | 16  (1.0812) | 19  (1.0339) | 7  (0.7735) |  | 49  (7.2891) | 58  (17.9766) | 83  (18.9296) | 51  (19.1138) |  | 9  (6.3970) | 15  (16.1596) | 20  (17.6578) | 26  (18.4545) |
| 5 | 5  (0.4389) | 10  (0.9767) | 14  (0.9105) | 15  (0.6911) |  | 43  (7.5460) | 72  (18.2498) | 86  (18.2871) | 62  (17.3726) |  | 5  (6.4435) | 16  (16.2696) | 23  (17.2027) | 16  (16.9333) |
| 6 | 3  (0.2994) | 3  (0.7089) | 10  (0.6200) | 8  (0.4204) |  | 23  (4.9080) | 40  (12.1650) | 44  (12.3787) | 31  (12.1177) |  | 5  (4.4016) | 13  (10.9829) | 13  (11.4301) | 8  (11.6829) |
