# Supplementary material for: Effectiveness of Seasonal Malaria Chemoprevention in Children under Ten Years of Age in Senegal: A Stepped-Wedge Cluster-Randomised Trial
Source: PLoS Med. 2016 Nov 22;13(11):e1002175. doi: 10.1371/journal.pmed.1002175 (PMC5119693; doi:10.1371/journal.pmed.1002175)
Supplement: S3 Table — (DOCX) [file pmed.1002175.s008.docx]

S3 Table Effect of SMC in each age group during the transmission season (from September 15 to December 15 each year) and when cases throughout the year were included (from Aug 1^st^ 2008, when surveillance started, to December 31^st^ 2008, and from January 1^st^ to December 31^st^ in 2009 and in 2010). Estimates are adjusted for effects of age, calendar year and zone. There was no evidence of an indirect effect in the first year of the study when SMC was implemented only in children under 5 years of age, the corresponding term was dropped from the models.

|  |  | Malaria confirmed  by RDT or slide | Cases treated for  malaria with or  without confirmation |
| --- | --- | --- | --- |
| During the transmission season | | Incidence rate ratio  (95%CI) | Incidence rate ratio  (95%CI) |
| Children 3-59 months | No SMC | 1 | 1 |
|  | SMC | 0.43 (0.37,0.52) | 0.28 (0.24,0.32) |
| Children 60-119 months | No SMC | 1 | 1 |
|  | SMC | 0.39 (0.33,0.45) | 0.35 (0.31,0.41) |
| Both age groups combined | No SMC | 1 | 1 |
|  | SMC | 0.40 (0.36,0.46) | 0.31 (0.28,0.35) |
| Indirect effects in older age groups | No SMC in the area | 1 | 1 |
|  | SMC in the area | 0.74 (0.67,0.82) | 0.72 (0.65,0.79) |
|  |  |  |  |
| Including cases reported thoughout the calendar year | | |  |
| Children 3-59 months | No SMC | 1 | 1 |
|  | SMC | 0.55 (0.47,0.63) | 0.37 (0.33,0.41) |
| Children 60-119 months | No SMC | 1 | 1 |
|  | SMC | 0.50 (0.44,0.57) | 0.45 (0.40,0.50) |
| Both age groups combined | No SMC | 1 | 1 |
|  | SMC | 0.52 (0.46,0.58) | 0.40 (0.37,0.44) |
| Indirect effects in older age groups | No SMC in the area | 1 | 1 |
|  | SMC in the area | 0.80 (0.73,0.88) | 0.80 (0.75,0.87) |

|  | <5yrs | | | 5-9yrs | | | 10-20yrs | | | 20+yrs | | |
| --- | --- | --- | --- | --- | --- | --- | --- | --- | --- | --- | --- | --- |
| Zone | 2008 | 2009 | 2010 | 2008 | 2009 | 2010 | 2008 | 2009 | 2010 | 2008 | 2009 | 2010 |
| 1 | 72 | 12 | 104 | 139 | 23 | 132 | 192 | 66 | 419 | 334 | 96 | 505 |
|  | 90 | 13 | 104 | 157 | 24 | 132 | 216 | 67 | 422 | 378 | 96 | 507 |
|  | 6.3775 | 6.48775 | 5.90825 | 4.44825 | 4.82825 | 5.00275 | 7.6785 | 7.9545 | 7.944 | 14.5405 | 15.35525 | 16.27575 |
|  |  |  |  |  |  |  |  |  |  |  |  |  |
| 2 | 43 | 2 | 19 | 60 | 8 | 33 | 109 | 52 | 172 | 88 | 47 | 214 |
|  | 72 | 2 | 20 | 70 | 9 | 34 | 129 | 56 | 182 | 109 | 48 | 222 |
|  | 6.409 | 6.3965 | 5.81375 | 4.21475 | 4.57625 | 4.755 | 6.48575 | 6.72275 | 6.79075 | 11.35175 | 11.77275 | 12.55825 |
|  |  |  |  |  |  |  |  |  |  |  |  |  |
| 3 | 73 | 12 | 30 | 58 | 13 | 53 | 87 | 51 | 191 | 127 | 84 | 265 |
|  | 175 | 12 | 30 | 120 | 13 | 53 | 135 | 51 | 192 | 233 | 84 | 266 |
|  | 5.095 | 5.08025 | 4.59925 | 3.57025 | 3.78675 | 3.90125 | 6.21475 | 6.3315 | 6.35625 | 11.46025 | 11.979 | 12.65725 |
|  |  |  |  |  |  |  |  |  |  |  |  |  |
| 4 | 40 | 13 | 20 | 32 | 26 | 61 | 81 | 89 | 285 | 93 | 113 | 354 |
|  | 112 | 16 | 21 | 62 | 26 | 66 | 144 | 92 | 323 | 172 | 117 | 405 |
|  | 5.7565 | 6.09175 | 5.435 | 3.96 | 4.474 | 4.59175 | 6.46525 | 7.0255 | 7.13 | 10.878 | 11.91275 | 12.70975 |
|  |  |  |  |  |  |  |  |  |  |  |  |  |
| 5 | 53 | 17 | 45 | 78 | 37 | 92 | 139 | 72 | 283 | 143 | 88 | 342 |
|  | 127 | 17 | 45 | 120 | 38 | 96 | 194 | 72 | 287 | 218 | 89 | 345 |
|  | 5.97 | 6.05 | 5.541 | 4.013 | 4.28475 | 4.443 | 6.64925 | 6.844 | 6.89 | 12.035 | 12.52725 | 13.31325 |
|  |  |  |  |  |  |  |  |  |  |  |  |  |
| 6 | 32 | 3 | 61 | 53 | 6 | 115 | 74 | 37 | 257 | 105 | 63 | 369 |
|  | 39 | 3 | 67 | 59 | 6 | 118 | 78 | 38 | 264 | 113 | 65 | 391 |
|  | 3.918 | 3.9615 | 3.5635 | 2.67175 | 2.80375 | 2.8845 | 4.4435 | 4.5815 | 4.61375 | 8.08625 | 8.4085 | 8.93125 |

Incidence of malaria during the transmission season. The number of confirmed cases (upper figure), number of cases treated, and person time at risk (1000s of person years) (lower figure) is given for each zone, age group and year.
