# Supplementary material for: Effectiveness of Seasonal Malaria Chemoprevention in Children under Ten Years of Age in Senegal: A Stepped-Wedge Cluster-Randomised Trial
Source: PLoS Med. 2016 Nov 22;13(11):e1002175. doi: 10.1371/journal.pmed.1002175 (PMC5119693; doi:10.1371/journal.pmed.1002175)
Supplement: S4 Table — (DOCX) [file pmed.1002175.s009.docx]

S4 Table Incidence of severe malaria

|  | No. of cases (1000’s of person years) | |  |
| --- | --- | --- | --- |
|  | SMC | Control | Rate ratio |
| Children under 5 years of age | | | |
| 2008 | 5 (5.9885) | 11 (24.2451) |  |
| 2009 | 6 (16.5034) | 12 (14.3277) |  |
| 2010 | 14 (27.8916) | 2 (3.6396) |  |
|  |  |  | 0.70 (0.36,1.4) P=0.326 |
| Children 5-9 years of age | | | |
| 2008 | - | 22 (26.5159) |  |
| 2009 | 8 (14.9780) | 11 (12.9837) |  |
| 2010 | 26 (26.1002) | 6 (3.3766) |  |
|  |  |  | 0.44 (0.23,0.84)P=0.013 |
|  | Both age groups combined: | | 0.55 (0.32,0.94)P=0.031 |
