# Supplementary material for: Effectiveness of Seasonal Malaria Chemoprevention in Children under Ten Years of Age in Senegal: A Stepped-Wedge Cluster-Randomised Trial
Source: PLoS Med. 2016 Nov 22;13(11):e1002175. doi: 10.1371/journal.pmed.1002175 (PMC5119693; doi:10.1371/journal.pmed.1002175)
Supplement: S5 Table — (DOCX) [file pmed.1002175.s010.docx]

S5 Table Prevalence of gametocyte carriage in children under 5 years old (2008) and under 10 years old (2009 and 2010)*, in SMC and control areas.

|  |  | Prevalence  (95% CI) | Prevalence ratio (95%CI) | P-value |
| --- | --- | --- | --- | --- |
| 2008 | Non-SMC area | 2.3% | 1 |  |
|  | SMC area | 0.64% | 0.28 (0.10, 0.75) | 0.012 |
| 2009 | Non-SMC area | 0.21% | 1 |  |
|  | SMC area | 0.11% | 0.51(0.12, 2.15) | 0.360 |
| 2010 | Non-SMC area | 0.41% | 1 |  |
|  | SMC area | 0.98% | 2.4 (0.3,19) | 0.400 |

*in 2008, children who were aged 3-59 months in September of that year; in 2009, and in 2010, children who were aged 3-119 months in September of that year.
