# Supplementary material for: Effectiveness of Seasonal Malaria Chemoprevention in Children under Ten Years of Age in Senegal: A Stepped-Wedge Cluster-Randomised Trial
Source: PLoS Med. 2016 Nov 22;13(11):e1002175. doi: 10.1371/journal.pmed.1002175 (PMC5119693; doi:10.1371/journal.pmed.1002175)
Supplement: S6 Table — (DOCX) [file pmed.1002175.s011.docx]

S6 Table Mean haemoglobin concentration at the end of the 2008 and 2009 transmission seasons in SMC and non-SMC areas.

|  |  |  | Mean (g/dL) | Difference (95%CI) |
| --- | --- | --- | --- | --- |
| 2008 | **Children under 5 years of age** | |  |  |
|  |  | No SMC | 9.78 | Reference |
|  |  | SMC | 9.92 | 0.14 (-0.07, 0.34) P=0.2 |
| 2009 | **Children under 5 years of age** | |  |  |
|  |  | No SMC | 9.87 | Reference |
|  |  | SMC | 9.79 | -0.07 (-0.25, 0.10)P=0.41 |
|  | **Children 5-9 years of age** | |  |  |
|  |  | No SMC | 11.11 | Reference |
|  |  | SMC | 11.15 | -0.03 (-0.13, 0.21)P=0.65 |
|  | **Both age groups combined** | |  |  |
|  |  | No SMC | 10.49 | Reference |
|  |  | SMC | 10.51 | 0.02 (-0.12, 0.15)P=0.81 |
