# Supplementary material for: Effectiveness of Seasonal Malaria Chemoprevention in Children under Ten Years of Age in Senegal: A Stepped-Wedge Cluster-Randomised Trial
Source: PLoS Med. 2016 Nov 22;13(11):e1002175. doi: 10.1371/journal.pmed.1002175 (PMC5119693; doi:10.1371/journal.pmed.1002175)
Supplement: S7 Table — (DOCX) [file pmed.1002175.s012.docx]

S7 Table The prevalence of moderate (Hb<11g/dL) and severe (Hb<6g/dL) anaemia at the end of the 2008 and 2009 transmission seasons in SMC and non-SMC areas.

|  |  |  | % prevalence | Prevalence ratio (95%CI) |
| --- | --- | --- | --- | --- |
| 2008 | **Children under 5 years of age** | |  |  |
|  | Moderate anaemia | No SMC | 29.3 | 1 |
|  |  | SMC | 27.1 | 0.92 (0.79,1.07) P=0.30 |
|  |  |  |  |  |
|  | Severe anaemia | No SMC | 1.07 | 1 |
|  |  | SMC | 0.32 | 0.30 (0.07,1.19) P=0.09 |
|  |  |  |  |  |
| 2009 | **Children under 5 years of age** | |  |  |
|  | Moderate anaemia | No SMC | 27.6 | 1 |
|  |  | SMC | 27.9 | 1.0 (0.86,1.15) P=0.97 |
|  | Severe anaemia | No SMC | 1.04 | 1 |
|  |  | SMC | 2.22 | 2.14 (0.93,4.9) P=0.08 |
|  | **Children 5-9 years of age** | |  |  |
|  | Moderate anaemia | No SMC | 9.2 | 1 |
|  |  | SMC | 8.9 | 1.0 (0.86,1.15) P=0.97 |
|  | Severe anaemia | No SMC | 1.20 | 1 |
|  |  | SMC | 1.21 | 1.01 (0.44,2.33) P=0.99 |
|  | **Both age groups combined** | |  |  |
|  | Moderate anaemia | No SMC | 18.4 | 1 |
|  |  | SMC | 17.9 | 0.97 (0.84,1.13) P=0.71 |
|  | Severe anaemia | No SMC | 1.12 | 1 |
|  |  | SMC | 1.69 | 1.51 (0.80,2.86) P=0.21 |
