# Supplementary material for: Effectiveness of Seasonal Malaria Chemoprevention in Children under Ten Years of Age in Senegal: A Stepped-Wedge Cluster-Randomised Trial
Source: PLoS Med. 2016 Nov 22;13(11):e1002175. doi: 10.1371/journal.pmed.1002175 (PMC5119693; doi:10.1371/journal.pmed.1002175)
Supplement: S8 Table — (DOCX) [file pmed.1002175.s013.docx]

S8 Table Rebound effects: Age-adjusted incidence rate ratios (confirmed malaria in the 2011 transmission season) in relation to the number of previous years of SMC.

|  | Incidence rate ratio (95%CI) | P-value |
| --- | --- | --- |
| No SMC | 1 |  |
| SMC for 1 year | 0.77 (0.59,1.01) | P=0.059 |
| SMC for 2 years | 1.09 (0.82,1.45) | P=0.531 |
| SMC for 3 years | 1.18 (0.84,1.67) | P=0.340 |
