# Supplementary material for: Effectiveness of Seasonal Malaria Chemoprevention in Children under Ten Years of Age in Senegal: A Stepped-Wedge Cluster-Randomised Trial
Source: PLoS Med. 2016 Nov 22;13(11):e1002175. doi: 10.1371/journal.pmed.1002175 (PMC5119693; doi:10.1371/journal.pmed.1002175)
Supplement: S1 Text — (DOC) [file pmed.1002175.s014.doc]

*Proposal – Summary Information*

| **Project Name:** | *Large-scale implementation of Intermittent Preventive Treatment in Children Delivered through Health Services with Community Participation in the Sahel* |
| --- | --- |

| **Disease/Health Condition/Issue Addressed:** | *Malaria Intermittent Preventive Treatment in Children* |
| --- | --- |

| **Organization Name:** | Département de Parasitologie - Faculté de Médecine – Université de Dakar, Sénégal |
| --- | --- |

###### Primary Contact for Proposal:

| Prefix | *Prof* | First name | *Oumar* | | Surname | *Gaye* | | *Suffix* |  |
| --- | --- | --- | --- | --- | --- | --- | --- | --- | --- |
| Last name | Gaye | | | Suffix | | |  | | |
| Title | *Prof* | | | Telephone | | | *+*221 825 19 98 | | |
| Address | *Department of Medical Parasitology, University of Dakar* | | | Fax | | | *+*221 825 36 68 | | |
|  | | | | | |
| E-mail | [*ogaye@refer.sn*](mailto:ogaye@refer.sn)*;* [*badara.cisse@lshtm.ac.uk*](mailto:badara.cisse@lshtm.ac.uk) | | | | | | | | |
| Web site | [*www.ucad.sn*](http://www.lshtm.ac.uk/) | | | | | | | | |

| **Amount Requested (U.S. dollars**): | | 3 964 546 | | --- | | **Project Duration (months):** | 36 |
| --- | --- | --- | --- | --- |
| **Does your project involve clinical trials with human subjects?** | | YES | |

| **U.S. Tax Status** *(Refer to* [*Tax Status Definitions*](http://www.gatesfoundation.org/GlobalHealth/Grantseekers/RelatedInfo/TaxStatusDefinitions.htm)*)*: | | **Non-U.S. Charitable Organization** |
| --- | --- | --- |
| **Geographic Location(s) of project:** | Senegal | |

**Charitable Purpose:** *Please include the following components: disease, health condition or issue;* [*strategic approach*](http://www.gatesfoundation.org/GlobalHealth/Grantseekers/grantmakingpriorities.htm)*; if appropriate, location of activity and limit to 255 characters.*

The purpose of this project is to reduce mortality and morbidity from malaria in Senegalese children by the administration of antimalarial drugs to children under the age of five years on three occasions during the high transmission season.

**Project Description** *(describe how the funds would be used to meet the charitable purpose, limit to 150 words)*:

The study will be undertaken in rural Senegal where mortality and morbidity from malaria are high in children. Sulfadoxine-pyrimethamine plus amodiaquine will be given to children under five by village-based community workers on three occasions during the high transmission season. The intervention will be introduced in a staggered way over three years, allowing evaluation of its impact. Approximately 100 thousand children in 725 villages will be included. Insecticide treated bednets will be made available throughout the study area at highly subsidized cost. The primary end point will be all causes mortality and the sample size has been set to meet this objective. Other clinical endpoints will be prevalence of parasitaemia and of anaemia at the end of the transmission season, and the incidence of hospital cases of severe malaria. Drug resistance will be monitored. Participation in this study will strengthen the ability of Senegalese scientists to undertake large-scale intervention studies.

**List of investigators**

- El H. Ba ^(1)^ (Parasitologist, Molecular Biology)
- Denis Boulanger^(1)^ (Immunologist, Immuno-Hematology)
- Badara Cisse ^(2, 3)^ (Clinical epidemiologist, Deputy Project Director, SOP, PI)
- Yemou Dieng ^(2, 4)^ (Professor of Clinical Parasitology, advisor)
- Fatou Ba Fall ^(4)^ (Research officer of the National Malaria Control Programme, co-coordinator implementation activities)
- Babacar Faye ^(2, 4)^ (Clinical Parasitologist, in vivo studies, Molecular Biology, Co PI)
- Oumar Faye ^(2, 4)^ (Professor of Clinical Parasitology, advisor)
- Oumar Gaye^(2, 4)^ (Professor of Clinical Parasitology, Head of the Department, Project Director)
- Brian Greenwood ^(2)^ (Director of the Gates malaria Partnership, advisor)
- Rachel Hallett^(2)^ (Molecular Biology, Training)
- Paul Milligan ^(2)^ (Epidemiologist/statistician, study design, data analysis)
- Daouda Ndiaye ^(2, 4)^ (Biologist, Molecular Biology)
- Jean-Louis Ndiaye ^(2, 4)^ (Clinical Parasitologist and Public Health, verbal autopsy review, field supervision Co PI)
- François Simondon ^(1)^ (Clinical epidemiologist, advisor)
- Kirsten Simondon ^(1)^ (Nutritionist, advisor)
- Cheikh Sokhna ^(1, 4)^ (Biologist, SOP, PI evaluation)
- Dembel Sow ^(2, 4)^ (Professor of Pediatrics, advisor)
- Colin Sutherland ^(2)^ (Molecular Biologist, advisor)
- Pape M. Thior ^(4)^ (Coordinator of the National Malaria Control Programme, coordinator implementation activities)
- Jean-François Trape ^(1)^ (Clinical Epidemiologist, advisor)

1. Institut de Recherche pour le Développement

2. Faculty of Medicine, University of Dakar

3. London School of Hygiene and Tropical Medicine

4. National Malaria Control Programme, Ministry of Health, Dakar, Senegal

**SUMMARY**

## Rationale

In the Sahel, malaria transmission is seasonal. The burden of malaria is limited to three months a year, from September to November. During this period, malaria mortality and morbidity are very high, not only in infants but also in children aged 1-4 years who are the most at-risk group.

In 2002, a randomized, placebo-controlled trial was undertaken in the Niakhar District of Sénégal, with support from the Gates Malaria Partnership (GMP). Sulphadoxine plus pyrimethamine (SP) and artesunate were given to children under the age of five years three times during the malaria transmission season. An 86% reduction in the incidence of clinical malaria was observed, suggesting that this new strategy could be a highly cost-effective approach for reducing the burden of malaria in the Sahel and in other parts of Africa with a short transmission season.

It is now important to determine to what extend seasonal intermittent preventive treatment of malaria in children (sIPTc) will work on a large scale. In this project, we propose to evaluate the effectiveness of sIPTc when delivered through routine health services with community participation.

## Aim

To improve control of malaria in areas of Africa where malaria transmission is strictly seasonal and where most malaria deaths occur in children aged 0-4 years.

## General Objective

- To assess the effectiveness of seasonal IPT when delivered through health services, with community participation.
- To evaluate the impact of sIPT in children on all-causes mortality.

## Specific Objectives

- To determine if sIPTc with sulfadoxine-pyrimethamine plus amodiaquine (SP/AQ) can be delivered effectively to children 0-4 years through routine health services with community participation.
- To evaluate the impact of sIPTc on all-cause mortality among children 0-4 years of age, in a population where ITNs are made available at highly subsidized cost.
- To evaluate the impact on the incidence of hospital cases of severe malaria.
- To evaluate the impact of the intervention on the prevalence of malaria parasitaemia and anaemia in children 0-4 years at the end of the transmission season.
- To investigate if the introduction of sIPTc lead to rapid changes in the drug sensitivity of *Plasmodium falciparum*
- To estimate the cost-effectiveness of introducing sIPTc for children 0-4 years in a setting where malaria treatment is through routine presumptive treatment with ACT.
- To investigate the influence of sIPTc delivered through the health services on preventive and / or treatment-seeking behavior for malaria.
- To determine the acceptability of sIPTc and the sustainability of delivery through community participation.

Study site

Bambey, Fatick and Mbour Medical Districts in Senegal.

## Study design

The study area will be divided into 48 intervention zones, each one corresponding to a public health post under the Ministry of Health and its catchment population. Approximately 725 villages will be included in the trial. Each health post covers an average of 15 villages. The total population in the study area is approximately 450,000 with 95,000 children aged 3 to 59 months.

In each village, a register of eligible children whose parents are willing to participate in the study will be established by the chief of the village under the supervision of the nurse in charge of the health post. For each village, two to six persons, according to the number of children enrolled in the trial, will be trained to give preventive treatment in September, October and November each year and to record on the register the children who received preventive treatment. The nurse who will be responsible for organizing community participation in the project will provide drugs in advance. Parents of children enrolled in the study will be encouraged to bring their children to the health post for treatment of any episode of fever that occurs during the period of the trial. Treatment of malaria attacks according to the National Guidelines will be available for these children at the health post.

Deaths occurring during the study will be reported in the register held in each village with a record of the date.

The implementation will be phased in gradually. In the first year, intervention will be conducted in 8 zones, in the second year in 24 zones, and in the third year in 40 zones, and in the 4^th^ year in all 48 zones. This design enables a statistically efficient and unbiased comparison of mortality rates with and without the intervention. The design involves the taking over of coordination of implementation by the Ministry of Health and the community gradually during the project.

To investigate the effectiveness of the intervention, the following measurements will be made:

- Proportion of eligible villages where a register of eligible children is held.
- Proportion of eligible villages, which participated fully in the study.
- Proportion of eligible villages where drugs for sIPTc were available at the proposed times of administration.
- Proportion of eligible villages where drugs for sIPTc were delivered.
- Proportion of eligible children who received one, two and three doses of sIPTc.
- Modalities of community participation and interactions with the health system and the project staff.
- Impact of sIPTc on the incidence of hospital cases of severe malaria.
- Deaths occurring during the transmission season in eligible children, which will be investigated by post-mortem questionnaire.
- Hemoglobin levels and parasitemia carriage after sIPTc will be investigated in a random sample of children.
- The prevalence of molecular markers of resistance of *Plasmodium falciparum* to sulfadoxine-pyrimethamine.
- The acceptability of the intervention and the involvement of the community implementation.
- Equitability of access to the intervention.

## Trial duration

From January 2007 to January 2010.

## Ethical considerations

Approvals will be sought from:

- The Ethics Review board of the Senegalese Ministry of Health.

- The Ethics Committee of the London School of Hygiene & Tropical Medicine.

**BACKGROUND AND RATIONALE**

1. Aspects of malaria control in Sub-Saharan Africa

Malaria is by far the most significant tropical disease. In Sub-Saharan Africa, it remains the most common cause of morbidity and mortality. Direct mortality is estimated to be between one and three million deaths a year; the vast majority of these deaths occur among young children, especially in rural areas (Snow *et al*., 2005, Breman *et al.*, 2001).

During the last two decades, the emergence and spread of chloroquine resistance has been associated with a dramatic increase in malaria mortality. Hospital-based studies and population-based studies conducted in various countries in Africa have documented a two- or three-fold increase in malaria deaths and admissions for severe malaria, an increase temporally related to the emergence of chloroquine resistance (Trape, 2001).

In 1993, Malawi became the first African country to change its national policy for the treatment of uncomplicated malaria from chloroquine to sulfadoxine-pyrimethamine. Many other countries have now modified their national policies. In order to prevent the rapid emergence of resistance to alternative drugs, there is now general agreement that combination therapy should be used for the treatment of malaria, as for tuberculosis and AIDs control programmes (White *et al*., 1999).

2. Malaria intermittent preventive treatment (IPT)

Malaria intermittent preventive treatment is a new concept. It can be defined as drug administration in curative doses with the objective of preventing malaria; the provision of the drug is irrespective of the presence of parasites or symptoms. According to WHO (WHO Experts Committee, 2001), specifications for malaria intermittent treatment include: (a) the drug used must significantly lower parasitemia, but not necessarily clear parasites (b) drugs used must be safe (c) there must be a good prospect of a pediatric formulation (d) the drug must not suppress the immune response to co-administered vaccines.

Three drug combinations were thought to meet these specifications: sulfadoxine-pyrimethamine plus artesunate (SP/AS), amodiaquine plus artesunate (AQ/AS), and lumefantrine plus artemether (Coartem). Of these, SP/AS was considered by a WHO meeting in 2001 to be the first choice for IPT. Recently we have completed a trial (Sokhna et al, unpublished data) of four alternative regimens for seasonal IPT in children, showing that SP plus three daily doses of amodiaquine (SP/AQ) is more effective than the artemisinin combinations SP/AS and AS/AQ. Use of SP/AQ for IPT would allow ACTs to be reserved for treatment of clinical cases where there rapid action will be particularly useful.

Two community trials have evaluated malaria intermittent treatment during infancy. In a district of Tanzania where malaria is holo-endemic, Schellenberg *et al.* (2001) observed that IPT administration with SP was associated with a reduction of 59% in clinical attacks of malaria and of 50% in severe anemia in infants. In this study, IPT was administered three times during the first year of life as part of the Expanded Programme of Immunization (EPI). In another study in Tanzania in which IPT (SP+amodiaquine) was given every two months, the efficacy of intermittent treatment in preventing malaria attacks in infants was 65% (Massaga *et al*. (2003). In Ghana, IPTi with SP led to a 26% reduction in clinical attacks of malaria (Chandramohan *et al.,* 2005). Further trials of IPT in infants are underway.

3. Seasonal intermittent preventive treatment of malaria in children (sIPTc)

Studies in Tanzania, where malaria transmission is intense and perennial, have shown that IPT during infancy has the potential to become a major tool for malaria control in highly endemic areas of Africa. In such areas, most deaths attributable to malaria occur during the first year of life and, in such circumstances, a pragmatic approach to delivery is to make use of the Expanded Programme of Immunization. IPT is a logical addition to this package.

However, the situation is completely different in many parts of Africa, perhaps with a population of around 250 million (Greenwood, unpublished), where malaria transmission is seasonal. In such areas, protective immunity is acquired slowly and only a small percentage of malaria attributable mortality occurs during infancy. Furthermore, since births occur all year round, most administrations of IPT given at the time of EPI will take place at a period of the year where there is no malaria transmission. This is particularly the case in the Sahel where malaria transmission lasts three months or even less and where about 80% of malaria deaths occur in children aged 1-4 years.

For these reasons, we proposed in 2002 a new approach for IPT in the Sahel, in which three administrations of treatment would be given monthly during the three months of the malaria season. We have evaluated this approach in the Niakhar area, Sénégal, with support from the Gates Malaria Partnership (GMP). From September to November 2002, a randomized, placebo-controlled trial was undertaken among 1,200 children under the age of five years. A single dose of sulfadoxine-pyrimethamine + artesunate was given to 600 children in September, October and November whilst, 600 other children received a placebo. Results showed a reduction in the incidence of clinical malaria of 86%, suggesting that this new strategy could be a highly cost-effective approach for reducing the burden of malaria in the Sahel and in other parts of Africa with a short transmission season. No rebound in the incidence of clinical malaria was seen in the recipients of sIPTc in the year after the intervention.

In this proposal, we will evaluate on a large scale, the effectiveness of sIPTc when delivered through routine health services with different levels of community participation.

**AIM, GOALS AND OBJECTIVES**

## GOAL

To reduce the burden of malaria in West Africa.

## AIM

To improve control of malaria in areas of Africa where malaria transmission is highly seasonal and where most malaria deaths occur in children aged 1-4 years.

GENERAL OBJECTIVE

To assess the effectiveness of sIPTc when delivered through the health services with community participation in a population in which ITNs are made available at highly subsidized cost.

SPECIFIC OBJECTIVES

- To determine if sIPTc with SP/AQ can be delivered effectively to children 0-4 years through the routine health services with community participation.
- To evaluate the impact of sIPTc on all-cause mortality among children 0-4 years of age.
- To evaluate as a secondary analysis the additional benefit of sIPTc on all cause mortality among children using ITNs.
- To evaluate the impact of sIPTc on the incidence of hospital cases of severe malaria and on the prevalence of anemia and malaria parasitaemia in children 0-4 years.
- To investigate whether the introduction of sIPTc with SP/AQ leads to rapid changes in the drug sensitivity of *Plasmodium falciparum* to SP
- To determine the cost-effectiveness of introducing sIPTc for children 0-4 years.
- To investigate the influence of sIPTc delivered through the health posts on preventive and treatment-seeking behavior for malaria.
- To determine the acceptability of sIPTc and the sustainability of delivery through community participation, and the equitability of access to the intervention.
- To assess the risk of rebound after IPT treatment is stopped.

**PROJECT DESIGN AND METHOD**

STUDY SITE

The study will take place in an area of 2816 km^2^ situated between the latitudes 14°20’N and 14°55’N, and the longitudes 16°17’W and 16°42’W. Approximately 450,000 persons live in this area (2002 national census). The northern part of the study area belongs to Bambey medical district and the southern part belongs to Fatick medical district. There are 725 villages in the study area (Figure 1). There are 48 health posts headed by a nurse and two health centers headed by a medical doctor. All health posts are under the direct supervision of the Senegalese Ministry of Health.

The study site is adjacent to, but does not include, the 30 villages of the Niakhar study area. The 31,000 inhabitants of this area have been under continuous Demographic and Health Surveillance System since 1984 and a considerable amount of data is available on this population including, in particular, malaria epidemiology and trends in all-cause and malaria-specific morbidity and mortality (Delaunay *et al*., 1998, Trape *et al*., 1998, Robert *et al*., 1998).

Figure 1: The study site (Medical Districts of Mbour, Bambey & Fatick)


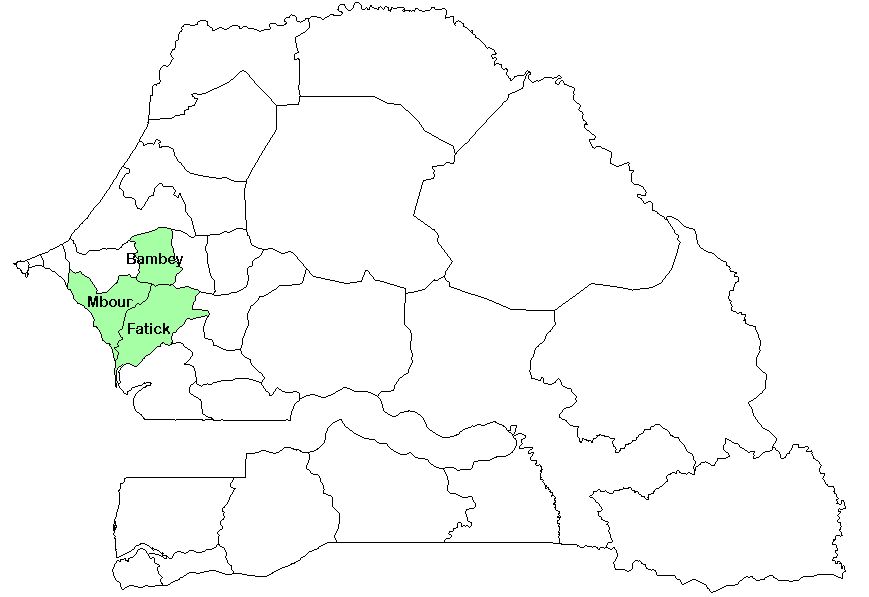


The Gambia

Mauritania

Atlantic ocean

Mali

Dakar

### SENEGAL

Guinea Bissau

Guinea Republic

TRIAL DESIGN

1. Study population

The study population will number approximately 450,000, including 95,000 children under the age of five years (2002 national census). The study area will be divided into 48 intervention zones, each one corresponding to one health post and its related villages. On average, 15 villages and 1975 children under five years of age will be the responsibility of each health post. The chief of the village, or a person designated by him, will establish a register of eligible children. This will be done under the supervision of the nurse in charge of the health post. This register will remain permanently in the village and a copy will be kept in the health post. Community consent will be obtained for the sIPTc intervention; individual consent will be sought from parents or guardians from a subgroup for participation of children in cross-sectional surveys.

Recent surveys conducted by NetMark show that coverage of Insecticide Treated Nets (ITNs) has increased in the study area and this is expected to increase with the widespread provision of ITNs at highly subsidized cost.

2. Design, sample size and power calculations

Due to logistic constraints and the need for staff training and community sensitization, it is not feasible to introduce the intervention in all zones simultaneously and it must be phased in gradually. This provides the opportunity for evaluation by comparing intervention areas with areas that have not yet received the intervention. The order of implementation will be randomized to permit unbiased comparison of mortality rates. To enable the most efficient evaluation, zones will be grouped into strata of zones such that mortality rates are as similar as possible in the zones within each stratum, using the best proxies available for the under 5 mortality rate. We plan phased introduction over 3 years, so there are three strata, the order of implementation of sIPTc in each stratum will be determined by randomization (Figure 2). The definition of the strata, and the order in which strata receive the intervention, will be decided in close consultation with the Ministry of Health. The project statistician will determine the order of implementation within strata.

Figure 2: Phased introduction of the implementation in the population divided onto 3 strata

Stratum A B C

Year 1 X0

Year 2 XX X0

Year 3 XX XX X0

Year 4 XX XX XX

Here “X” represents a group of 8 zones (16,000 children 3-59 months) receiving intervention, “0” represents a group of 8 zones that are kept under malaria and mortality surveillance but which have not yet received the intervention. For groups “0”, intervention is delivered the following year. Thus, intervention is gradually phased in, in the first year we will intervene in 8 zones, in the second year in 24 zones, and in the third year in 40 zones, and in the 4^th^ year in all 48 zones. This design enables a statistically efficient and unbiased comparison of mortality rates with and without the intervention. In year 4, implementation will be taken over by the Ministry of Health and the community. Rebound effects in children over 5 yrs will be assessed through a case control study.

Prior to the implementation study, a four-months survey will be conducted to estimate mortality rates under children five years in the three districts of the study area. This cross sectional study will also determine socio economic predictors of areas with a high mortality.

2.1. Pilot cluster sample survey to determine under five mortality rates in the 3 districts of the study area

The sample size and design of the study rely on good estimates of childhood mortality rates. Excellent estimates are available from the Niakhar area from demographic surveillance, but we do not know the situation in the other parts of the study area. In addition, for efficient evaluation of the impact of sIPTc on mortality it is important that we stratify health zones into strata of similar mortality rate before randomization. However, correlates of higher mortality on which stratification could be based are not well known, this survey will provide an opportunity to determine these correlates. Lastly, the survey will allow us to estimate the coefficient of variation of mortality rates between zones, an important quantity in the sample size calculation for the randomized implementation study.

We will therefore conduct a survey to estimate mortality rates among children under 5 years in the three districts of the study area, and determine socioeconomic predictors of areas of high mortality.

The area will be divided into four strata for survey purposes, these will be:

1. The area of the Niakhar DSS.
2. The remaining part of Fatick district not covered by the DSS.
3. Bambey district (excluding DSS).
4. Mbour district.

Making one survey stratum coincide with Niakhar DSS will allow us to compare survey estimates of mortality with actual estimates determined from the DSS and adjust the estimates accordingly. We will use the methods for mortality estimation described in the UNICEF publication on measuring childhood mortality (David et al 1990) (the Brass method, birth histories, and preceding birth technique). Within strata we will select zones (the primary sampling unit) with probability proportional to size, and within zones select households for interviewing all women aged 15 to 49 years to ask about birth histories and deaths. Making the primary sampling units of the mortality survey coincide with the clusters (zones) used for randomization of the sIPTc implementation will allow us to estimate the design effect applicable to the randomized intervention. Female interviewers will be used. The sample size will be calculated carefully but for provisional estimation of the budget we anticipate that we will need to interview 2000 women in each stratum, a total sample of 6000 interviews, and the budget reflects this.

2.2. Sample size for all-cause mortality

The sample size has been calculated using mortality data and verbal autopsy results from Niakhar, and hospital based data from the Ministry of Health for Thiès district, in order to be able to demonstrate an impact on all-cause mortality.

The 10-year average mortality rate among children 3-59 months in Niakhar is about 35 per 1000 child years. However, there has been a steady decline in all-cause mortality over time so a 10-year average is a poor predictor of the mortality we expect in the 4 years of our project. In addition, the average over the last 5 years has been influenced by a major epidemic of meningitis in 1998-2000.

The mortality rate in the age group 3 months to 5 years over the last 10 years in Niakhar is as follows (Niakhar DSS database, IRD, Dakar):

Year No. Deaths Person years Rate per child per year

1993: 193 4852 0.0398

1994: 136 4859 0.0280

1995: 173 4872 0.0355

1996: 174 4939 0.0352

1997: 141 5002 0.0282

1998: 335 4971 0.0674

1999: 288 4752 0.0606

2000: 187 4802 0.0389

2001: 140 5017 0.0279

2002: 134 5149 0.0260

2003: 122 5443 0.0224

In view of the decreasing trend of mortality rates over this period, and to allow for effects of an increase in coverage of ITNs, we have assumed an all-cause mortality rate of 0.02 (20 per 1000) as the most appropriate estimate to use for sample size calculations. The percentage of deaths caused by malaria as determined by verbal autopsies in 1993-2001 was 29% in children < 5years in the Niakhar DSS area (Niakhar DSS report). This percentage has increased over time while the overall all-cause mortality rate has decreased. Verbal autopsies have limited sensitivity and specificity, but the true proportion of deaths due to malaria can be estimated if the sensitivity and specificity are known, using the formula

Ptrue = (Pobs + specificity - 1) / (specificity + sensitivity - 1)

Korenromp et al. (Lancet Infectious Diseases 2003, 3:349) quote the sensitivity and specificity of verbal autopsies (VAs) for malaria deaths in Niakhar as 55% and 87% respectively. Adjusting for the sensitivity and specificity of the VAs technique implies a true fraction of 38% of deaths due to malaria is obtained. We have some confidence in this figure because it agrees well with the percentage of deaths in hospital that had malaria as the primary cause of death. From Ministry of Health statistics, the percentage of deaths in hospital due to malaria in the whole country among children under 5 yrs was 35% in 2000. For the Thies district, the percentages of malaria deaths under 5 years during 1997-2000 were:

1997: 37%

1998: 23%

1999: 27%

2000: 42%

The drop in the percentage of deaths due to malaria in 1998-9 is likely to be due to the meningitis epidemic, which caused a large number of additional deaths in 1998 and 1999. Thus, we have taken the true fraction of malaria deaths to be 38% based on the corrected VA data. This may be a slightly conservative estimate because it is a 10-year average and malaria-specific mortality has been increasing during this period.

If sIPTc has 90% efficacy against malaria deaths, we anticipate a 0.38 x 0.9=34% drop in all cause mortality due to sIPTc. If we assume a 70% efficacy, (reflecting incomplete coverage of 3 doses when sIPTc is delivered by routine health services rather than by trial staff) the drop would be 27%. If we have 70% efficacy and the percentage of malaria deaths turns out to be lower than we anticipate, say 30%, the drop in all cause mortality would be 21%. We have done sample size calculations for these three scenarios. Furthermore the sample size also depends on the degree of variability between zones in the all-cause mortality rate. This is usually expressed as the coefficient of variation, k. We do not have site-specific estimate of k but, for a wide range of outcomes in cluster-randomized trials, k is usually in the region of 0.25 to 0.3 (Hayes and Bennett, 1999). The table below presents sample size calculations for these scenarios, firstly assuming a coefficient of variation of 0.25, and then repeated assuming the coefficient of variation is 0.3. All calculations assume there is an average of 2000 children 3 months to 5 years in each zone.

Mortality rate True Efficacy of % reduction No. of zones for:

per 1000 % due to sIPTc against in all-cause 90% power 80% power Description

child years malaria malaria deaths mortality

k=0.25

20 38% 70% 27% 22x2=44 16x2=32 Most likely scenario

20 30% 70% 21% 35x2=70 26x2=52 Lower % malaria deaths

25 30% 70% 27% 20x2=40 15x2=30 Higher all cause mortality

k=0.30

20 38% 70% 27% 27x2=54 20x2=40 Most likely scenario

20 30% 70% 21% 45x2=90 34x2=68 Lower % malaria deaths

25 30% 70% 27% 26x2=52 20x2=40 Higher all cause mortality

Therefore a sample size of 44 zones would give us 90% power if k=0.25 and 80% power if k=0.3, in 2 of the 3 scenarios. In the worse case scenario of low all cause mortality and lower than expected % malaria deaths, the power is more limited (44 zones would give us 61% power if k=0.3 and 72% power if k=0.25). If the all-cause mortality is higher than we predict, say 35 per 1000, which is the 10-year average figure, the worst case scenario, with 44 zones, has 78% power if k=0.25 and 65% power if k=0.3. If the all-causes mortality is 20 per 1000, but efficacy of IPT against malaria deaths is 90%, then the power for the worse case, with 44 zones, is 90%.

The study population is therefore about 95,000 children 3-59 months of age living in the 48 interventions zones.

Power calculations will be updated using themost recent data from Niakhar DSS, when these become available. An interim analysis will be done at the end of year 2; conditional power calculations will be done to in order to check the adequacy of the projected sample size.

2.3. Sample size rationale for the cross-sectional surveys in December for parasitology, parasite resistant genotypes, anemia, febrile children and malnutrition endpoints:

***Sample selection****:* 150 children aged 3 months to 5 years will be selected from each cluster (zone) for testing in December. Blood will be obtained for hemoglobin, thick smear for microscopy, and parasite genotyping, and assessment of nutritional status will be made. In each zone, children will be selected using a 2-stage cluster sampling design. Communities will be selected with probability proportional to size, and a fixed number of children selected from each selected community from the village register by systematic sampling.

***Anemia and parasitemia prevalence endpoints:*** Assuming a coefficient of variation between clusters of 0.25, this sample size indicate above will give 80% power (using a 5% significance level and allowing for 20% loss to follow up) to detect, in the first year of the study, a halving in the prevalence of a) anemia (hemoglobin<8 g/dL) which has an expected prevalence of 15% in control clusters); b) parasitemia prevalence (expected prevalence of 20% in control clusters). Combining the data for the three years (strata) will give power for smaller magnitude effects; c) prevalence of fever with parasitaemia in children.

***Prevalence of SP resistant parasite genotypes:*** Another endpoint of interest is the prevalence of drug resistant genotypes in December. We will determine the prevalence of the DHFR triple mutation and the DHPS (codon 437) mutation associated with resistance to pyrimethamine and sulfadoxine respectively. We have calculated power for non-inferiority, to show that IPT does not make SP resistance worse, and the power to detect an important reduction in prevalence of SP resistance genotypes associated with sIPTc.

We anticipate that 37% of samples will be positive for asexual parasitemia in the control arm and 13% in the IPT arm (based on figures from the Niakhar study) in December. Assuming 90% of these samples yield DNA for parasite genotyping, and assuming the prevalence of the DHFR triple mutation is 75% in the controls, we would need in an *individually* randomized study, in order to be able to rule out, using a standard one-sided test of non-inferiority (90% power, one-sided alpha level of 0.05), the possibility that the prevalence of the DHFR triple mutation has increased by more than 10% in the IPT group, 257 parasite positive samples in each arm. However this would require over sampling in the IPT arm, taking 695 from the control group and 1977 from the sIPTc arm; if we prefer to sample equally in both arms we would need 448 parasite positive children in the control arm and 158 in the sIPTc arm. This would mean sampling 1342 from each arm. This would be simpler because the same samples could be used for the other endpoints. However, these figures must be increased to allow for cluster randomization. Assuming the intra class correlation coefficient is about 0.01 (equivalent to assuming the prevalence of the triple mutation ranges from zone to zone from 65% to 85%), then the design effect (DE, which measures the proportionate increase in sample size that is required) can be calculated for a given sample size per cluster, DE=(1+(m-1)ρ) (where m is the mean number per cluster and ρ is the intra cluster correlation coefficient). If m=150, the DE is about 2.5, and we will need 1342x2x2.5=6684 children to be sampled in total, which will be achieved by the end of year 3 if we sample 150 children per cluster (zone) each year. For the second case described in the preceding paragraph, i.e. the calculations for the case where we are interested in the superiority of IPT in terms of reducing prevalence of SP resistant mutations, we have 90% power to detect a reduction of the prevalence of the triple mutation from 75% in the control arm to 60% in the sIPTc arm, at the end of year 2 if we sample 150 children in each zone in each year. A secondary endpoint will be the overall prevalence of resistant genotypes (using all sampled children as denominator, rather than just the parasite positive children).

***Malnutrition:*** 30% of children under 5 years in the control arm of the Niakhar sIPTc study were malnourished (weight for age z-score less then 2 SD below the median of the reference population), and 26% in the sIPTc group (Cisse unpublished data). Effective malaria prevention may be expected to have an impact on nutritional status by preventing weight loss during disease episodes. The study will have 90% power (using a 5% significance level) to detect a reduction in the prevalence of malnutrition from 30% to 20%, assuming a coefficient of variation of 0.35 (we believe malnutrition may be more variable from cluster to cluster than some of the other endpoints), if we include 150 children in the December surveys each year from each cluster.

2.4. Malaria morbidity

Malaria cases will be treated at health centres, where treatment is with AQ/AS based on presumptive clinical diagnosis.,

3. Drug administration

Delivery of sIPTc will be the responsibility of the National Malaria Control Programme. It is planned that in each village two to six persons will be trained to give preventive treatment to children on 1^st^ September, 1^st^ October and 1^st^ November, or on the nearest appropriate day, and to record in the register the children who received preventive treatment. The community will designate these persons and the nurse will be responsible for their training in correct drug administration. The nurse will provide drugs in advance to the chief of the village. Deaths occurring during the trial will be recorded immediately in the register with a record of the date of death. The operational details will be piloted in 2005. Children will be issued with a malaria card similar to the vaccination card bearing their name and ID and on which treatments will be recorded.

The choice of drug combination to be used in the main trial is SP/AQ, based on the results of a study undertaken in Niakhar in 2004 in which 2102 children were randomized to receive sIPTc with one of four drug combinations (1) SP x 1+ artesunate x 1; (2) SP x 1 + artesunate x 3; (3) SP x 1 + amodiaquine x 3 and (4) amodiaquine x 3 + artesunate x 3. SP/AQ was significantly more effective in terms of malaria incidence and parasite prevalence (Sokhna et al., in preparation), and its use allows artemisinins to be reserved for treatment of clinical malaria cases where their rapid action will be of most benefit. A report of this trial has been submitted to the BMGF, and a paper has been prepared for publication.

4. Community participation

The minimum community participation required from villages benefiting from the trial will be the identification of persons to keep the register and to give preventive treatment to children.

In order to stimulate additional community participation for the maximum effectiveness of the trial, a special budget will be allocated to each intervention zone under the responsibility of study nurse. He or she will decide, in agreement with the community leaders, how this budget could be best used for public health education and advocacy. This will be noted on a specific form.

5. Treatment of episodes of illness

Parents or guardians of children enrolled in the study will be encouraged to bring their children to the health post for any episode of illness that occurs during the trial. Antimalarial treatment with AQ/AS will be provided by government staff in health posts according to the national guidelines. Children who present with an acute febrile illness with no other obvious cause of the illness will be treated presumptively for malaria. All consultations of children, their symptoms on presentation and treatments received will be noted in a register.

6. Phased introduction of the intervention

***- Administration of sIPTc***

In the first year of the trial, registers of eligible children will be established in Stratum A comprising 16 zones. sIPTc will be administered in only 8 of these zones, selected at random. Throughout the study area antimalarial treatment according to the national guidelines will be available in the health post (Figure 3).

In the second year of the trial, the intervention will be extended to the remaining 8 zones of Stratum A and also it will be introduced in 8 new zones in Stratum B.

In the third year of the trial, intervention will be extended to the remaining 8 zones of Stratum B and to 8 new zones in Stratum C.

In each stratum, implementation will be taken over by the Ministry of Health and the trial communities in the second year of implementation.

**Figure 3**: A simplified model of the study design and diverse types of comparison.

***- Age at inclusion***

The minimum age for inclusion in the trial will be three months. All other children under five years of age will be eligible for the trial. In the second and the third year of the trial, children previously enrolled in the trial but who have reached their fifth birthday will be excluded from sIPTc.

7. Scaling-up ITN coverage in the study area

It will be important to ensure that ITN coverage in the study area is high in order to be able to demonstrate that IPTc provides significant additional protection in children using ITNs. It is likely that the effects of the two interventions will be additive. In a split-plot design trial in Sierra Leone, villages were randomised to receive ITNs or no nets, and within each village children were randomized to receive prophylaxis with dapsone / pyrimethamine or placebo once every 2 weeks. The effects of the exclusive use of either ITNs or prophylaxis on protection against clinical malaria due to P. falciparum was similar (49% and 42%, respectively), while in combination this protective efficacy was significantly increased to 72% (Marbiah et al. 1998). Assuming additive effects, we expect IPTc, which has efficacy of 90-95% in recent efficacy trials in Niakhar, will reduce malaria incidence and malaria mortality by this percentage in children using ITNs.

ITN coverage in the study area has increased in recent years. Surveys conducted by NetMark in 2000 and 2004 in Dakar, Thies, Saint Louis, Kaolack and Tambacounda showed that the number of households that owned a net increased from 34% (2000) to 56% (2004). In 2004, 24% of children under 5 years of age slept under an ITN the night before the survey. In Mbour district, there is no recent assessment of the use of nets, but with the intervention of CCF/Canah, the coverage rate is expected to be high. The peripheral market is dominated by highly subsidized ITNs sold in the health structures (health centres or health posts). In 2005, very few nets were distributed free. Four types of ITN share the Senegalese market (3 conventional and one long lasting Permanet) distributed through pharmaceutical companies and the private sector. To maximise ITN coverage the National Malaria Control Programme in collaboration with UNICEF will provide treated nets at substantially subsidized cost from 2006, and the project will facilitate the distribution of ITNs at the community level by working in close collaboration with local partners. In the last coordination’s meeting held at the University of Dakar in June 2005, it had been suggested that the Minister of Health form a steering committee, which will oversee all IPTc implementation studies in the country. This steering committee would also help to prioritise the study area for scaling up ITN coverage in collaboration with UNICEF, WHO, JICA, USAID and NGOs activities and through the National Malaria Control Programme. Several technical meetings are being held with the senior staffs of the Ministry of health, or the coordinator of the National Malaria Control Programme and with the local representative of UNICEF in Dakar.

ASSESSMENT OF THE IMPACT OF THE INTERVENTION

1. Process measurements

The following measurements will be made each year:

**- Proportion of eligible villages where a register of eligible children is held.**

Criterion used: presence of a register in the village (all 725 villages investigated).

**- Proportion of eligible villages, which participated fully in the study**.

Criterion used: number of villages in which at least 50% of the total number of resident children according to the results of the 2002 national census have been included in the registers (all villages investigated). Reasons for non-participation will be investigated through community focus group discussions.

**- Proportion of eligible village where drugs for sIPTc were available on the 1st September, 1st October and 1st November each year.**

Criterion used: presence of drugs for sIPTc at these three dates in villages with sIPTc (all 725 villages investigated according to year).

**- Proportion of eligible villages where drugs for sIPTc were delivered.**

Criterion used: presence in the village on 1^st^ September, 1^st^ October and 1^st^ November of persons trained to give preventive treatment to children (all 725 villages investigated) and evidence of administration of drugs to at least 50 % of the children mentioned in the register in villages receiving sIPTc. Reasons for logistical problems will be followed up through interviews with those involved**.**

**- Proportion of eligible children who received one, two and three doses of sIPTc.**

Criteria used: (1) children who received one, two and three doses of sIPTc as indicated in the register; (2) presence of sulfadoxine-pyrimethamine in the capillary blood of a sample of 300 eligible children (sampling during the two first weeks of September, October and November; children will be selected using a cluster survey design with villages selected with probability proportional to size. The parents of children who did not receive the full three doses will be interviewed to find out the reasons for default. This part of the study will be done using similar methods to those developed for surveys of vaccination coverage.

**- equitability of access to the intervention in relation to health and wealth indicators**

To determine equitability of access to the intervention in terms of health, wealth, and access to other interventions, we will record ITN use, vaccination history, nutritional status, and household assets for determining wealth quintile during the cluster sample survey.

**- Modalities of community participation and interactions with the health system and the project staff*.***

The respective roles of the nurse in charge of each health post and of the project staff in charge of a given intervention zone and of the community, in the success or the failure of sIPTc in each given village will be evaluated. In-depth interviews will be held to obtain detailed information on the factors that contributed to either success or failure. In addition to the previous criteria, other quantitative or qualitative criteria will be used, including monthly number of consultations, monthly number of infants vaccinated and the proportion of the population covered by the health post, availability of essential drugs in the health post and approaches used to improve participation in sIPTc and its linkage with routine activities in general, and EPI in particular. For 10 villages with maximum participation in sIPTc and 10 villages with low or no participation in sIPTc, additional investigations will include focus group discussions and participant observation at the village level.

2. Measurement of the impact of the intervention

**- All cause mortality and malaria-specific mortality**

All deaths occurring in eligible children during the year will be investigated by the verbal autopsy method with the same questionnaire and procedures used since 1984 for the sites of Niakhar, Mlomp and Bandafassi in Sénégal (Trape *et al.* 1998). In each village we will identify reporters who will record any death of a recruited child in the study area in a register. Study staff will also visit each village on a monthly basis to check the registers. A verbal autopsy will be completed for each recorded death within one month following the death using a standardized questionnaire (Garenne and Fontaine, 1986 (unpublished)). Non-medical interviewers who are members of the community and speak the local language will make household visits. Two physicians will sequentially review each verbal autopsy questionnaire. Cause of death will be attributed and followed by an independent assignment of ICD-9 codes. If the two physicians disagree, a panel of physicians involved in the Senegalese Working Group will discuss the case and reach a consensus diagnosis. If no consensus can be reached the cause will be recorded as unknown.

**- Incidence of hospital cases of severe malaria**

The area is served by two small district hospitals (Bambey and Mbour). There are referral hospitals at Thies and Fatick. Hospital admissions with severe malaria (i.e. malaria that presents with life threatening conditions, including coma, severe anaemia, renal failure, respiratory distress syndrome, hypoglycaemia, shock, spontaneous haemorrhage, and convulsions) will be documented. Records will be kept of admission of study children. Physicians and nurses based at these facilities will check whether children with severe malaria live in the study area, and record carefully details from their malaria ID card, and clinical biochemical and parasitological data will be recorded on a Case Report Form, with treatment details, final diagnosis using standard WHO criteria, and outcome.

**- Prevalence of anemia and parasitemia**

Hemoglobin measurements using a *Hemocue* machine, and finger prick sampling for thick smears for parasite density determination, will be done once in each stratum, at the end of transmission season, in a sub-sample of children. For these endpoints, 150 children will be randomly selected from each cluster. A slide will be declared negative only after 200 high power fields are examined without finding a parasite. A second independent laboratory technician will reread all collected slides. If parasite densities recorded by the two readers differ by more than 30%, a third senior laboratory technician will be asked to adjudicate. Anemia will be defined by hemoglobin level of less than 8 g/dL.

**- Prevalence of clinical malaria**

Impact on clinical malaria will be determined through limited passive surveillance, used to estimate the proportion of fever cases that have malaria, and by determining the prevalence of clinical malaria, defined as fever with asexual parasitaemia above a cut-off of 3000/ul, in the cross-sectional surveys in December.

**- Nutritional status**

Nutritional status will be measured each year at the end of the transmission season, in a random sample of 150 children per cluster. Weight for age index will be used as an estimate of the prevalence of malnourished children.

**- Drug sensitivity of *P. falciparum***

In order to assess the impact of the intervention on the sensitivity of *P. falciparum* to antimalarials, *in vivo* tests and molecular markers studies will be undertaken.

For sulfadoxine-pyrimethamine resistance investigations, molecular markers studies (DHFR and DHPS) will be conducted each year at the community levels, before and after the intervention. In addition to the thick smear, a drop of blood will be taken on a filter paper in a random sample of children at the end of the malaria season transmission., Markers of resistance to pyrimethamine and sulfonamides will be detected using a PCR assay as previously described (Pearce et al. 2003)

For evaluation of sIPTc for all these endpoints, data from Stratum A in year 1, Stratum B in year 2 and Stratum C in year 3 will be used in a stratified analysis comparing zones with and without sIPTc (Figure 4).

3. Safety studies

**- Toxicity**

Both sulfadoxine-pyrimethamine, amodiaquine and artemisinin derivatives have low toxicity and are widely used in malaria endemic countries for the curative, presumptive and/or preventive treatment of malaria among infants, children, pregnant women and adults. Artemisinin based combination therapy is recommended by WHO for treatment of malaria. SP plus. amodiaquine has until recently been the first line antimalarial in Senegal (now changed to AQ/AS) and has been widely used in this population.

In each health post, the nurse will hold a register where all cases of side effects possibly attributable to the trial drug treatment will be noted. Nurses will be taught to look out for severe skin lesions that might be associated with SP administration. A physician will investigate any reports. If severe side effects are suspected, a specific form will be filled and immediately transmitted to the Data and Safety Monitoring Board (DSMB). In October and December each year, the Principal Investigator (Intervention) of the project will transmit to the DSMB a synthesis of data collected by the nurse on the possible mild side effects attributable to sIPTc.

Figure 4: Stratified analysis in Stratum A in year 1, Stratum B in year 2 and Stratum C in year 3

**Stratum A** **Stratum B** **Stratum C**

|  |  |  |  |  |  |  |  |  |  |  |  |  |  |  |  |  |  |  |  |  |  |  |
| --- | --- | --- | --- | --- | --- | --- | --- | --- | --- | --- | --- | --- | --- | --- | --- | --- | --- | --- | --- | --- | --- | --- |
| Year 1 |  |  |  |  |  |  |  |  |  |  |  |  |  |  |  |  |  |  |  |  |  |  |
|  |  |  |  |  |  |  |  |  |  |  |  |  |  |  |  |  |  |  |  |  |  |  |
|  |  |  |  |  |  |  |  |  |  |  |  |  |  |  |  |  |  |  |  |  |  |  |
| Year 2 |  |  |  |  |  |  |  |  |  |  |  |  |  |  |  |  |  |  |  |  |  |  |
|  |  |  |  |  |  |  |  |  |  |  |  |  |  |  |  |  |  |  |  |  |  |  |
|  |  |  |  |  |  |  |  |  |  |  |  |  |  |  |  |  |  |  |  |  |  |  |
| Year 3 |  |  |  |  |  |  |  |  |  |  |  |  |  |  |  |  |  |  |  |  |  |  |
|  |  |  |  |  |  |  |  |  |  |  |  |  |  |  |  |  |  |  |  |  |  |  |
|  |  |  |  |  |  |  |  |  |  |  |  |  |  |  |  |  |  |  |  |  |  |  |
| Year 4 |  |  |  |  |  |  |  |  |  |  |  |  |  |  |  |  |  |  |  |  |  |  |
|  |  |  |  |  |  |  |  |  |  |  |  |  |  |  |  |  |  |  |  |  |  |  |
|  |  |  |  |  |  |  |  |  |  |  |  |  |  |  |  |  |  |  |  |  |  |  |
|  |  | = 2 zones (4000 children) where sIPTc is implemented | | | | | | | | | | | | | | | | | | | | |
|  |  |  |  |  |  |  |  |  |  |  |  |  |  |  |  |  |  |  |  |  |  |  |
|  |  | = 2 zones (4000 children) where sIPTc non yet implemented | | | | | | | | | | | | | | | | | | | | |
|  |  |  |  |  |  |  |  |  |  |  |  |  |  |  |  |  |  |  |  |  |  |  |

**- Rebound effect**

It will be important to compare malaria incidence after the age of 5 in children who received IPT with those that did not receive IPT, in order to determine whether sIPTc impairs acquisition of natural protective immunity. This will be done through a case control approach, cases will of two types, all deaths in children in the study area, and all hospital cases of severe malaria, among children over 5yrs, but young enough to have been eligible for IPT. For each case, controls will be sought, for cases and controls IPT doses received will be determined from the malaria card and cross checked against the study database, and the risk of death or severe disease related to the duration of IPT treatment prior to age 5. Cases and controls will be recruited in years 2-4 in stratum A, years 3-4 in stratum B, and year 4 in stratum C.

4. Costs effectiveness studies

The methodology will follow an incremental cost-effectiveness approach with the drugs being delivered through communities in the intervention zones. Cost-effectiveness Ratios (CERs) will be estimated for each condition in terms of cost per death and cost per hospital case of severe malaria averted.

A broader societal perspective will be taken with emphasis not only on the costs to the providers but also both direct and indirect costs for patients, their caregivers and the community.

Tools for data collection

A set of standard instruments will be used, similar to those used in other IPT studies. So far the following tools have been earmarked for adaptation and use in the Senegalese study:

- A data sheet for recording IPTi/sIPTc delivery costs at the district level.

- A data sheet for recording management and joint costs of the health system, reflecting both trial protocol and real program conditions.

- A set of data sheets for recording (expected) health system savings due to less malaria morbidity, based on the number of clinical cases avoided, health seeking patterns, and health service unit costs.

- A questionnaire for recording patient costs, through interviews.

Sample size requirements

In accordance with the guidelines of the CEWG, the sample size will be determined based on an ability to show an economic difference where one exists, to 95% of confidence. Sample sizes will be calculated for:

- Health system savings – i.e. a sample of the number of health facilities providing health care to study patients.

- Patient costs and savings – i.e. a sample of the infants receiving sIPTc and benefiting from less illness.

Proposed economic and Health outcomes

Table 1 shows the expected data to be collected, covering both cost and health outcomes. The study will collect costs on the delivery of sIPTc at the operational level, as well as cost offsets and patient costs (CEWG, 2004). Since the study is also interested in a real policy change and all the processes that go with a policy change, these costs will also be measured and presented. Inclusion of these costs is also important in a cost-effectiveness analysis, especially where such costs are significant and persist over time (CEWG, 2004).

# Table 1. Summary of proposed economic and health outcomes for cost-effectiveness analysis.

| Proposed cost outcomes to be included | | | | Health outcomes measured | | |
| --- | --- | --- | --- | --- | --- | --- |
| Policy change | Delivery of sIPTc | Cost offsets***** | Patient & community | Morbidity | Mortality | DALY |
| **√** | **√** | **√** | **√** | **√** | **√** | **√** |

*Source (CEWG)*

***** These consist mainly of costs saved due to less treatment seeking resulting from less malaria morbidity

**√** Indicates these data are to be measured prospectively for the given study.

5. Impact on health seeking behaviors

It is possible that sIPTc may affect treatment-seeking behavior for malaria, for example if it is misinterpreted as providing long-term protection similar to that of vaccines. As a result, adequate treatment could be delayed. Conversely, increased awareness of malaria as a result of sIPTc may enhance use of other preventive and curative measures. It is therefore important to know how people perceive sIPTc, and how it influences health behavior. A number of case studies will be made of episodes of childhood febrile illness to determine the influence of the intervention on treatment seeking behaviors. In order to place this in a broader context, community focus group discussions will be carried out to investigate people’s understanding of malaria, their treatment priorities, and their perceptions of the intervention. As the use of ACT may also influence people’s interpretations of both malaria and sIPTc, their treatment seeking behavior and their response to the introduction of the intervention will also be studied.

As the long-term success and sustainability of this intervention depend on the adequate functioning of a system of community participation, the interaction between health services, community representatives involved in the intervention and the target population will be studied using qualitative methods, in order to understand problems that arise and to allow the identification of potential impediments to implementation.

TIME TABLE

**Baseline survey and implementation study preparatory phase**

1^st^ July 2006 – 31 March 2007

Intervention

1^st^ April 2007 – 31 August 2007 preparatory phase 2

1^st^ September 2007 – 31 November 2007 intervention year 1

1^st^ December 2007 – 31 August 2008 preparatory phase 2

1^st^ September 2008 – 31 November 2008 intervention year 2

1^st^ December 2008 – 31 August 2009 preparatory phase 3

1^st^ September 2009 – 31 November 2009 intervention year 3

Evaluation

1^st^ April 2007 – 31 August 2007 preparatory phase

1^st^ September 2007 – 31 August 2008 evaluation year 1

1^st^ September 2008 – 31 August 2009 evaluation year 2

1^st^ September 2009 – 30 June 2010 final evaluation

**PROJECT MANAGEMENT AND DISSEMINATION PLAN**

The grant holding institution will be the University of Dakar, Department of Medical Parasitology, headed by Professor Oumar Gaye. The two co-principal investigators will assist the two principal investigators.

A “Project Advisory Committee” will be created with representatives of the Senegalese Ministry of Health and Prevention, the heads of the medical regions of Fatick, Thies and Diourbel, WHO and UNICEF. This group will meet every three months.

Finally, a “Project Directorial” will be formed. It will include the investigators listed in page 2. Selected members of this group will meet on a monthly basis, or more frequently if needed.

DATA AND SAFETY MONITORING

The trial will have a Data and Safety Monitoring Board (DSMB), consisting of members with clinical, statistical and local knowledge. Their responsibilities will be as follows:

1. To insure the investigators’ compliance with the study protocol

2. To monitor the progress of the trial (i.e. data and safety concerns of the study)

3. To make recommendations on further progress of the study

The DSMB members will meet once a year in Dakar, preferably in August, just before the drug administration starts. A second meeting each year, by conference call, is also envisaged. All members of this board will be paid by the project.

A local safety monitor designated by the DSMB will regularly visit the site.

ETHICS

As decided at the Dar es Salaam meeting in February 2005, communal informed and written consent will be obtained from trusted and respected community leaders. The study will explain the need to find an efficient way for large-scale prevention of malaria in children less than five years of age, using sIPTc.

Ethical approvals of the study will be sought from both:

- The Ethics Committee of the Senegalese Government

- The Ethics committee of the London School of Hygiene and Tropical Medicine.

**ORGANISATIONAL CAPACITY**

# UNIVERSITY OF DAKAR - SENEGAL

L'Université Cheikh Anta Diop (UCAD), located a few hundred yards from the Atlantic Ocean, is the oldest university in West Africa. Created during the French colonial era as the School of Higher Studies (L'Ecole de Haute Etudes), UCAD served all of French West Africa and contributed to the training of the first intellectuals, medical doctors, and academics in the region. The institution was later renamed the Université de Dakar and then L'Université Cheikh Anta Diop after one of Africa's most prolific and respected historians whose works (such as Civilization or Barbarism) note the parallels and brings into question the various cultural influences between "Black African" civilization and Egyptian civilization. Today, UCAD is Senegal’s largest university and is reputed to be the finest in francophone Africa. Some 43000 students from all of West Africa attend classes on this lively and friendly campus; it is not uncommon to see students from Sierra Leone in lively conversation with others from Mauritania and America. This sIPTc implementation project would mean strengthening of research activities on malaria, which are done with international standards. It would also provide a unique opportunity of training a pool of medical students of on field research.

# FRENCH RESEARCH FOR DEVELOPMENT INSTITUTE - SENEGAL

The IRD is a French public science and technology research institute under the joint authority of the French ministries in charge of research and overseas development. The IRD has three main missions: research, consultancy and training. It conducts scientific programs contributing to the sustainable development of the countries of the South, with an emphasis on the relationship between man and the environment.

# LONDON SCHOOL OF HYGIENE AND TROPICAL MEDICINE – UNITED KINGDOM

The London School of Hygiene and Tropical Medicine (LSHTM) was founded in 1899. Its charter received royal approval in 1924. LSHTM celebrated its centenary year in 1999. LSHTM’s mission is to contribute to the improvement of health worldwide through the pursuit of excellence in research, postgraduate teaching and advanced training in national and international public health and tropical medicine, and through informing policy and practice in these areas.

**BUDGET JUSTIFICATION**

1. Institutional fees

A rate of 10% of the requested budget, excluding equipment fees, has been used for overheads.

2. Personnel

For this implementation study, human resources will be decisive. An experienced, professional and well-motivated team of field-physicians, nurses and field workers is needed. At times, about sixty (60) local staff will be involved simultaneously in the field. All payments have been worked out according to the Senegalese salary scale.

Assistance of an IT manager is fundamental for design of the data management system, supervision of data entry and safekeeping of the data.

3. Equipment

For this study, good logistical support will be essential, as during the rainy season, the environment is unfavorable with poor quality of roads, power failures etc... Reliable 4X4 cars, motorbikes and generators are required for both implementation and evaluation teams. It is also planned to provide all the 48 health posts with basic medical equipment that will help to improve the health care delivered to all-those who attend. The offices of the study in Dakar will be equipped with basics tools such as computers and accessories.

4. Travel

For any member of the project directorial or any recruited consultant, the project will pay the cost of all journeys related to coordination, supervision, training or presentation of results.

5. Coordination and training

Coordinating such a trial will need a staff that is fully committed to the project. This will be done by the PIs. The project coordinator (full time PI) is a clinical epidemiologist who has overseen a number of Gates Malaria Partnership projects, in the capacity of project leader in Senegal. All these projects were very well managed and successfully completed. In addition, he has completed a Master of Sciences at the LSHTM in 2001. He successfully completed in September 2005 his PhD and is now based at the University of Dakar (Department of Medical Parasitology and Mycology) as a visiting lecturer.

The project will represent a unique opportunity for several young Senegalese scientists to gain experience in field research and implementation of large-scale programme to control malaria. Sadly, this area of research is presently neglected in many developing countries. This must be a point of great concern.

6. Mortality survey in year 1

The survey will be conducted immediately after the end of the 2006 malaria season, preliminary mapping from October 2006 and house-to-house interviews from December 2006 to March 2007, with data processing and statistical analysis continuing until June 2007. We will require 10 teams of two field workers and 3 supervisors, with additional costs for mapping, printing questionnaires, data entry, computers and software, logistic support, and consultancy visits from a survey specialist. Alternative funding has been sought for this survey and we therefore do not request funds for this in the present budget,

**CITATIONS**

Breman J.G. (2001). The ears of the hippopotamus: manifestations, determinants and estimates of the malaria burden. Am. J. Trop. Med. Hyg., 64 (Suppl): 1-11.

Chandramohan D. (2005). Prevention of malaria in infants by intermittent preventive treatment in an area of high, seasonal transmission in Ghana. BMG; 331; 727-733

David, PH, Bisharat, L and Hill, AG (1990) Measuring Childhood Mortality: a guide for simple surveys. UNICEF, New York.

Delaunay V. et al. (1998). La situation démographique et épidémiologique dans la zone de Niakhar au Sénégal. ORSTOM, Dakar.

Marbiah N.T., et al. (1998). A contolled trial of lambda-cyhalothrin-impregnated bednets and/or dapsone/pyrimethamine for malaria control in Sierra Leone. Am. J. Med. Hyg., **58**(1), , pp. 1-6.

Massaga, J.J., et al. (2003) Effect of intermittent treatment with amodiaquine on anemia and malarial fevers in infants in Tanzania: a randomised placebo-controlled trial. Lancet, **361**(9372): p. 1853-60

Pearce, R. J., C. Drakeley, et al. (2003). "Molecular determination of point mutation haplotypes in the dihydrofolate reductase and dihydropteroate synthase of *Plasmodium falciparum* in three districts of northern Tanzania." Antimicrob Agents Chemother **47**(4): 1347-54.

Robert V. et al. (1998). La transmission du paludisme dans la zone de Niakhar, Sénégal. Trop. Med. Int. Hlth, 3: 667-677.

Sauerborn R. et al. (1991). Estimating the direct and indirect economic cost of malaria in a rural district of Burkina-Faso. Trop. Med. Parasit., 42: 219-223.

Schellenberg D. et al. (2001). Intermittent treatment for malaria and anemia control at time of routine vaccinations in Tanzanian infants: a randomised, placebo controlled trial. Lancet, 357: 1471-1477.

Snow, R. W., C. A. Guerra, et al. (2005). "The global distribution of clinical episodes of Plasmodium falciparum malaria." Nature **434**(7030): 214-7.

Trape J.F. et al. (1998). Impact of chloroquine resistance on malaria mortality. C.R. Acad. Sci. (Ser. III), 321: 689-697.

Trape J.F. (2001). The public health impact of chloroquine resistance in Africa. Am. J. Trop. Med. Hyg., 64: 12-17.

White N. et al. (1999). Averting a malaria disaster. Lancet, 353: 1965-1967.

WHO (2001). Antimalarial Drug Combination therapy. WHO: Geneva.

WHO Experts Committee (2001). Preliminary meeting on malaria intermittent treatment in infants. WHO: Geneva.

# APPENDIX 1: Record form for inclusion & follow-up

______________________

District number |___|___| Village name & number |___|___|___|

Household owner Hamlet name:

# Mother name Mother’s ID Number: |___|___|___|___|___|___|

Mother age |___|___| Mother occupation

##### *Mother educational background* |___| (0=no, 1=primary, 2=secondary or more, 9=info no available)

# Child name: ID |___|___|___|___|___|___| - |___| Age |___|___| Sex |___|

|  | June/July  Ethics | August  Screening | September  1^st^ sIPT_c_ dose | October  2^nd^ sIPT_c_ dose | November  3^rd^ sIPT_c_ dose | December  Evaluation |
| --- | --- | --- | --- | --- | --- | --- |
| Date of visit |  |  |  |  |  |  |
| Auricular temperature | NA[[1]](#footnote-2)* |  |  |  |  |  |
| Drugs administered at home/health facility | NA | NA |  |  |  |  |
| Haemoglobin (g/dL) | NA | NA | NA | NA | NA |  |
| MPs/Per 200WBC | NA | NA | NA | NA | NA |  |
| Filter paper sample | NA | NA | NA | NA | NA |  |
| Weight (Kg) | NA |  | NA | NA |  | NA |
| Height (cm) | NA |  | NA | NA |  | NA |
| Mid arm circumference | NA |  | NA | NA |  | NA |
| Drugs retention (Y/N) | NA | NA | NA |  |  |  |
| Action taken if vomited | NA | NA |  |  |  | NA |

COMMENTS**:……………………………………………………………………………….…………………………………………………………………………………………………………………………………………………………………………………………………………………………………………………………………………………………………………………………………………………………………………………………………………………………………………………………………………………………………………………………………………………………………………...**

APPENDIX 2: Information to the parents or legal guardians

__________________________

We invite your child or ward XX to take part in a research study sponsored by the Faculty of Medicine, Pharmacy and Odonto-Stomatology (FMPOS) of the University of Sénégal, the French “Institut de Recherche pour le Développement” and the London School of Hygiene and Tropical Medicine. It is important that you understand several general principles that apply to all who take part in this study:

1. Participation in the study is entirely voluntary
2. You may withdraw your child from the study at any time
3. Personal benefit to your child will remain even after withdrawal from the study

Malaria is a sickness caused by a very small parasite that can get into the body when a mosquito bites you. It can cause fever, headaches, body aches and weakness, and if it is not treated, it can make a person severely ill, especially children. When malaria is treated with correct medicines, it can be cured completely, but some malaria parasites learn how to become resistant to these medicines. Therefore, we are having more and more problems for preventing or curing malaria.

The purpose of this study is to evaluate the effectiveness of a new approach that has been already shown very effective in preventing clinical malaria called sIPTc.

The reason that we invited your child to participate in this study is because we would like to learn about the advantages and limitations of IPT and about the best method to provide IPT to children under 5 years of age.

The alternative to participating in this study is to not participate.

If you agree that your child/ward participates in this study, for 3 years, we will provide free sIPTc . He/she may be selected for a finger prick at commencement or at the end of every rainy season. With this sample, we will try to find out information about things in your body (quality of blood, presence of parasite etc…).

The risks associated with this study are minimal. Finger prick may be a little painful but this should be greatly minimized as it will only be performed by well-trained nurses. The malaria treatment we provide is the same as that recommended by the Senegalese National Malaria Control Programme. The drugs used are associated with few adverse events such as skin disorders, dizziness etc…More details will be added when the trial drug combination has been selected. These adverse reactions will be closely monitored. It’s worth taking the antimalarial drugs as it has been proven that there are all effective in preventing or curing a clinical attack of malaria. Use of these medicines may also impact even on the transmission of malaria within the whole community.

If you wish to withdraw your child from the study, you may do so at any time without any negative consequences he or she has as participant. The findings of this study may be reported at meetings or in medical journals, but your child/ward’s name will not be used in the report, and the specific information we learn about you will not be shared with anybody except the study investigators.

Do you have any questions about your participation in this study?

If you have questions or concerns about your child/ward’s participation in this study at a later date, you may speak with your health post nurse, or you can ask your village responsible or one of the field supervisors to send word to Dr. Badara Cissé or Professor Oumar Gaye at the Faculty of Medicine, Pharmacy and Odonto-Stomatology in Dakar. The doctors at the medical districts can help you to directly contact Drs. Cissé or Gaye.

APPENDIX 3: Malaria episodes: clinical & laboratory data at days 0, 3, 7 & 14

#### _______________________________

Child name: ID |___|___|___|___|___|___| **-** |___| Age |___|___| Sex |___|

# Mother name:

District number |___|___| Village name & number |___|___|___|

Household owner: Hamlet name:

| Day of Protocol | D0 | D3 | D7 | D14 |
| --- | --- | --- | --- | --- |
| Date |  |  |  |  |
| Time |  |  |  |  |
| Temperature |  |  |  |  |
| Pulse |  |  |  |  |
| Respiratory Rate |  |  |  |  |
| Spleen Size |  |  |  |  |
| Parasitemia |  |  |  |  |
| Gametocytemia |  |  |  |  |
| Hemoglobin |  |  |  |  |

| Day of Protocol | D0 | D3 | D7 | D14 |
| --- | --- | --- | --- | --- |
| Fever/chills (Y/N) |  |  |  |  |
| Headache (Y/N) |  |  |  |  |
| Vomiting (Y/N) |  |  |  |  |
| Lethargy/abnormal consciousness (Y/N) |  |  |  |  |
| Convulsions (Y/N) |  |  |  |  |
| Respiratory distress (Y/N) |  |  |  |  |
| Able to eat or drink (Y/N) |  |  |  |  |
| Other (Y/N)  -  - |  |  |  |  |

APPENDIX 4: Serious adverse event report form

### ___________________

Child name: ID |___|___|___|___|___|___| **-** |___| Age |___|___| Sex |___|

# Mother name:

District number |___|___| Village name & number |___|___|___|

Household owner: Hamlet name:

| Date of serious adverse event: | | / / | |
| --- | --- | --- | --- |
| Location of sae (e.g., at home, health post or elsewhere): | |  | |
| Was this an unexpected adverse event? | | yes [ ] no [ ] | |
| Brief description of subject (do not include identifiers.) | | sex: m/f age:  diagnosis: | |
| Brief description of the nature of the serious adverse event: | | | |
| Category (outcome) of the serious adverse event:  [ ] death  [ ] life threatening  [ ] hospitalization  [ ] persistent or significant disability/incapacity  [ ] congenital anomalies or birth defects  [ ] other conditions which in the judgment of the investigators represent significant hazards | Relationship of serious adverse event to research:  [ ] 1 = unrelated (clearly not related to the research)  [ ] 2 = unlikely (doubtfully related to the research)  [ ] 3 = possible (may be related to the research)  [ ] 4 = probable (likely related to the research)  [ ] 5 = definite (clearly related to the research) | | |
| Have similar adverse events occurred on this protocol? | yes [ ] no [ ]  if “yes”, how many?  please describe: | | |
| What steps do you plan to take as a result of the adverse event reported above? provide documentation to the irb for review and approval of any of the steps checked below. | [ ] no action required  [ ] amend consent document  [ ] amend protocol  [ ] inform current subjects  [ ] terminate or suspend protocol  [ ] other, describe: | | |
| Investigator’s name & signature: | | | Date: / / |

# APPENDIX 5: Documentation of termination of participation in the study

______________________

Child name: ID |___|___|___|___|___|___| **-** |___| Age |___|___| Sex |___|

# Mother name:

District number |___|___| Village name & number |___|___|___|

Household owner: Hamlet name:

Participation of the volunteer in the study has been terminated because (check only one box):

🞏 The parents’ volunteer want to stop participating in the study

Reason

________________________________________________________________________

________________________________________________________________________

________________________________________________________________________

🞏 The study monitor believes it is not in the best interest of the child to continue in the study.

Reason

________________________________________________________________________

________________________________________________________________________

________________________________________________________________________

The parents have been informed of withdrawal and the justification for withdrawal

Signature of the Investigator _________________________________ Date ______

APPENDIX 6: summarized budget (in us $)

|  | **2007 & 2008** | **2009** | **2010** | **Total** |
| --- | --- | --- | --- | --- |
| Total Personnel | 496 215,00 | 612 735,00 | 729 255,00 | 1 838 206,00 |
|  |  |  |  |  |
| **Total Travel** | 22 000,00 | 22 000,00 | 22 000,00 | 66 000,00 |
|  |  |  |  |  |
| **Total Equipment** | 321 330,00 | 207 300,00 | 254 800,00 | 783 430,00 |
|  |  |  |  |  |
| **Medical and Laboratory** | 24 000,00 | 76 000,00 | 120 000,00 | 216 000,00 |
|  |  |  |  |  |
| **Other Supplies** | 10 500,00 | 10 500,00 | 10 500,00 | 31 500,00 |
|  |  |  |  |  |
| **Total Consultants** | 21 000,00 | 21 000,00 | 21 000,00 | 63 000,00 |
|  |  |  |  |  |
| Subtotal of Direct Costs | **895 045,00** | **945 535,00** | **1 157 555,00** | **2 998 135,00** |
|  |  |  |  |  |
| Overheads | 102 541,00 | 119 921,00 | 151 333,00 | 373 795,00 |

# APPENDIX 7: Letter from Prof. Awa Marie Coll Seck, then Ministry of Health


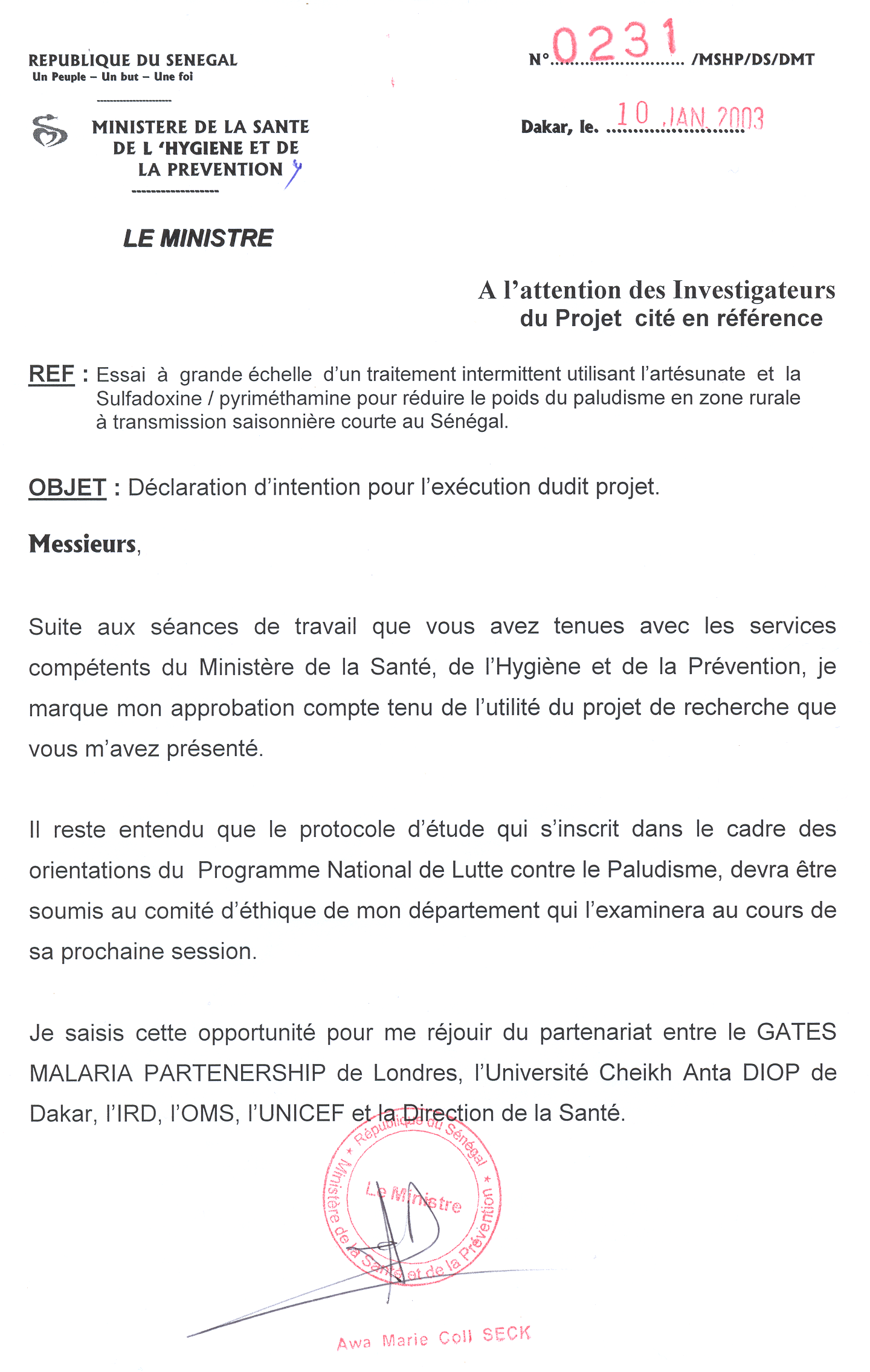
______________________

# APPENDIX 8: CVs of principal investigators

______________________

### 1) Badara Cisse, 44, Senegalese (PI & coordinator)

### MD, MSc, PhD

### Clinical epidemiologist

## Training

- Visiting clinical epidemiologist at the University Cheikh Anta Diop of Dakar (UCAD)

- PhD in Public Health Medicine at the London School of Hygiene and Tropical Medicine, London 2005

- Master in Epidemiology at the LSHTM, 2001

- Diploma on medical statistics and applied computer - Université Libre de Bruxelles, Brussels 1999

- C.E.S.A.M.[[2]](#footnote-3)1 Diploma – University Paris VI, 1994

- Pasteur Institute Epidemiology Diploma - Paris 1993

- Doctorate in Medicine - Dakar 1991

## Professional experiences

- Since October 2005, visiting senior clinical epidemiologist at the University of Dakar

- 2002 – 2003: Principal Investigator of a randomised double blind, controlled trial on seasonal IPT using artesunate plus sulfadoxine-pyrimethamine among 1203 children under five years of age.

- 1994 to 2000: Research assistant at the Research for Development Institute (IRD), Dakar.

- 1993: Trainee in epidemiology and public health at the Pasteur Institute, Paris.

- June 1991 to December 1994: Research assistant at the Pasteur Institute, Dakar.

- 1989 to 1990: Clinician on two-years voluntary service in rural Sénégal

###### Scientific works

- Ntab B., Simondon K., Milet J., Cisse B., Sokhna C., Boulanger D., Simondon F. A young child feeding index is not associated with height for age or height velocity in rural Senegalese children: American Society for Nutritional Sciences. American Society for Nutritional Sciences. 2005. J Nutr: 3 457-64.

- Remoué F., Cissé B., Ba F., Sokhna C., Boudin C., Hervé JP, Boulanger D. and Simondon F. Antibody response to Anopheles salivary antigens is a marker of risk for malaria. Trans. Roy. Soc. Trop. Med. Hyg. (2006). 100: 363-370

- Cisse B., Sokhna C., Boulanger D.,Milet J., Bâ EH., Hallett R., Sutherland C., Simondon K., Simondon F., Alexander N., Gaye O., Targett G.A.T., Lines J., Greenwood B.M., Trape J.F. Seasonal intermittent preventive treatment with artesunate and sulfadoxine pyrimethamine prevents malaria in Senegalese children. 2005: The Lancet, 2006. 367: 659-67

- Aaby P., Jensen H., Samb B., Cisse B., Sodemann M., Jakobsen M., Poulsen A., Rodrigues A., Lisse I. M., Simondon F., Whittle H. Differences in female-male mortality after high-titre measles vaccine and association with subsequent vaccination with diphtheria-tetanus-pertussis and inactivated poliovirus: reanalysis of West African studies: Lancet, 2003. 361(9376): p. 2183-8.

- Aaby P., Simondon F., Samb B., Cisse B., Jensen H., Lisse I. M., Soumare M., Whittle H., Low mortality after mild measles infection compared to uninfected children in rural West Africa. Vaccine, 2002. 21(1-2): p. 120-6.

- Aaby P., Whittle H., Cisse B., Samb B., Jensen H., Simondon F., The frailty hypothesis revisited: mainly weak children die of measles: Vaccine, 2001. 20(5-6): p. 949-53.

- Rogier C., LY A..B., Tall A., Cisse B, Trape J.F.Plasmodium falciparum clinical malaria in Dielmo, a holoendemic area in Sénégal: no influence of acquired immunity on initial symptomatology and severity of malaria attacks. Am J Trop Med Hyg, 1999. 60(3): p. 410-20

- Cissé B., Aaby P., Simondon F., Soumaré M., and Whittle H. The role of school in the transmission of measles in rural Sénégal: implications for its control in developing countries. Am J Epidemiol 1999 Feb, 15; 149(4)°: 295-301

- Bennett J., Whittle H., Samb B., Markowitz L., Simondon F., Cissé B., Aaby P. Seroconversions in unvaccinated infants: evidence of possible subclinical measles from vaccine trials in Niakhar, Sénégal. Int. J. Epidemiol, 1999. 28 (1): p. 147-51

- Whittle H., Aaby P., Samb B., Cissé B., Kanteh F., Soumaré M., Jensen H., Bennett J., Simondon F. Poor serologic responses 5-7 years after immunisation with high titre vaccines in early infancy. Pediatr Infect Dis J 1999 Jan; 18 (1): 53-7

- Samb B, Simondon F, Cissé B, Soumaré M, Bennett J, Markowitz L, Whittle H, Aaby P. Increased long-term mortality with rash following early measles vaccination in rural Sénégal. Pediatr Infect Dis J 1999 Jan; 18(1): 48-52

- Seng R., Samb B., Simondon F., Cisse B., Soumare M., Jensen H., Bennett J., Whittle H., Aaby P. (1999). "Increased long term mortality associated with rash after early measles vaccination in rural Sénégal." Pediatr Infect Dis J 18(1): 48-52.

- Cissé B, Aaby P, Whittle H, Samb B, Soumaré M, Bennett J, and Simondon F. Return of adult measles in Africa: is it more severe in the era of immunisation? Am J Epidemiol (in revision)

- Whittle H, Aaby P, Samb B, Cissé B, Bennett J, Simondon F. The importance of sub-clinical measles in maintaining immunity in vaccinated children in West Africa. Lancet (submitted)

- Aaby P, Cissé B, Simondon F, Samb B, Soumaré M, and Whittle H. Waning of vaccine-induced immunity: is it a problem in Africa? Am J Epidemiol 1999 Feb, 15; 149(4)°: 304-5

- Rogier C, Brau R, Tall A, Cissé B, Trape JF. Reducing the oral quinine-quinidine-cinchonine (Quinimax) treatment of uncomplicated malaria to 3 days does not increase the recurrence of attaks among children living in a highly endemic area of Sénégal. Trans. R. Soc. Trop. Med. Hyg. (1996) 90, 175 -178

- Diagne N, Rogier C, Cissé B, Trape JF. Incidence of clinical malaria in pregnant women exposed to intense perennial transmission. Trans. R. Soc. Trop. Med. Hyg. (1997) 91, 166-170

- Sidibe, E.H., Sow, A. M., Sarr, A., Cisse, B., Diop, S. N., Ka-Cisse, M., Bah, M. D., [Diabetic pregnancy in Sénégal (years 1980 to 1989)]. Ann Endocrinol (Paris), 1994. 55(5): p. 191-6

2) Cheikh-Sadibou Sokhna, 43, Senegalese

Professional address: UR 077 Paludologie Afrotropicale

IRD, POB 1386 CP 18 524 Dakar - Senegal

Tel. (221) 849 35 84 Fax. (221) 832 43 07

E-mail : [sokhna@ird.sn](mailto:sokhna@ird.sn)

###### TRAINING

2000: PhD, Département de Biologie Animale, Faculté des Sciences et Techniques, Université Cheikh Anta Diop de Dakar, Sénégal

1994: Master in Animal Biology, Département de Biologie Animale, Faculté des Sciences et Techniques, Université Cheikh Anta Diop de Dakar, Sénégal

# ADDITIONAL TRAINING

April 1999: Training on the International School WHO/EC/World Bank on "Tropical disease research Malaria: From basic research to planning control trials", University of Camerino, Italy.

February 1997: Training workshop "On design and methodology of malaria vaccine trials" organised by African Malaria Vaccine Testing Network (AMVTN), Ifakara, Tanzanie.

### POSITION AND RESPONSIBILITIES HELD

- Head of the Malaria team in Research Unit 077 and Local responsible of IRD of the Dielmo/Ndiop Project.

- As a member of Task force on the Senegalese National Malaria Control Programme.

EXPERIENCE IN THE CONDUCT OF CLINICAL TRIALS

- Co-Principal Investigator: A double blind randomised placebo-controlled trial to measure the potential of intermittent treatment with artesunate plus sulfadoxine/pyrimethamine (SP) to reduce the malaria burden in Sub-Saharan Africa.Impact of such an intervention on immunity after routine vaccinations. DFID and GMP 2002.

- Co-Principal Investigator: Surveillance for a potential rebound in malaria morbidity one year after intermittent presumptive treatment (IPT) in children in Niakhar, Senegal combined with a trial of two anti-malarial chemotherapeutic combinations. GMP 2003.

- Principal Investigator: A double blinded, randomised trial to compare the efficacy, safety and tolerability of four potential drugs regimens for sIPTc in Niakhar, rural Senegal. GMP 2004.

10 SELECTED PUBLICATIONS

SOKHNA C.S., MOLEZ J.F., NDIAYE P., SANE B. & TRAPE J.F. Tests in vivo de chimiosensibilité de Plasmodium falciparum à la chloroquine au Sénégal: évolution de la résistance et estimation de l’efficacité thérapeutique.Bulletin de la Société de Pathologie Exotique, 1997, 90, 2, 83-89.

## SOKHNA C.S., ROGIER C., DIEYE A., & TRAPE J.F. Host factors affecting the delay of reappearance of Plasmodium falciparum after radical treatment among a semimmune population exposed to intense perennial transmission., American Journal of Tropical Medicine and Hygiene, 2000, 62 (2), 266-270.

SOKHNA C.S., TRAPE J.F. & ROBERT V. Gametocytemia in children with falciparum malaria attack treated with chloroquine, amodiaquine or sulfadoxine+pyrimethamine. Parasite, 2001, 8, 243-250.

SOKHNA C.S., FAYE F.B.K., SPIEGEL A., DIENG H. & TRAPE J.F. Rapid reappearance of Plasmodium falciparum after drug treatment among Senegalese adults exposed to moderate seasonal transmission. American Journal of Tropical Medicine and Hygiene, 2001, 65 (3), 167-170.

DIAGNE N, ROGIER C, SOKHNA CS, TALL A, FONTENILLE D, ROUSILHON C, SPIEGEL A, TRAPE JF. Increased susceptibility in malaria during the early postpartum period. New. Engl. J. Med., 2000, 343: 598-603.

SOKHNA C.S., LE HESRAN J.Y., MBAYE P.A., AKIANA J., CAMARA P.I., DIOP M., LY. & DRUILHE P. Increase of malaria attacks among children presenting concomittant infection by Schistosoma Mansoni in Senegal. Malaria Journal. 2004. 3 : 43.

BACAER N & SOKHNA CS. A mathematical model representing the diffusion of resistance to an anti-malarial drug. Mathematical Biosciences and Engineering. 2005, vol 2. 227-238.

TALMAN AM, PAUL REL, SOKHNA CS, DOMARLE O, ARIEY F, TRAPE JF, ROBERT V. Influence of chemotherapy on the Plasmodium gamétocyte sex ratio of Mice and human. Am J Trop Med Hyg. 2004. 71: 739-744.

REMOUE F, CISSE B, BA F, SOKHNA CS, HERVE JP, BOULANGER D & SIMONDON F. Evaluation of the antibody response to Anopheles salivary antigens as a potential marker of risk for malaria. Transactions of Royal Society of Tropical Medicine an Hygiene, 2006, 100, 363-370..

CISSE B, SOKHNA CS, BOULANGER D, MILET J, BA EH, RICHARDSON K, HALLET R, SUTHERLAND C, SIMONDON K, SIMONDON F, ALEXANDER N, GAYE O, TARGETT G, LINES J, GREENWOOD B & TRAPE JF. Seasonal intermittent preventive treatment with artesunate and sulfadoxine-pyrimethamine for prevention of malaria in Senegalese children: a randomised, placebo-controlled, double-blind trial. The Lancet, 2006. 367: 659-667.

**Statistical Analysis Plan**

**Dec 2013**

Outcomes:

All-cause mortality in children aged 3-59 months and 60-119 months (primary outcome)

Cause of deaths in 13 health post areas where verbal autopsies will be performed

Incidence of RDT-confirmed malaria

Incidence of malaria (confirmed and unconfirmed)

Incidence of severe malaria (admission to hospital with primary diagnosis of malaria)

IPTc Coverage: proportion who received 1,2 and 3 courses of IPTc, and the proportion who received no IPTc, each year of the study

ITN Coverage

Prevalence of parasitaemia at the end of each transmission season

Prevalence of gametocyte carriage

Prevalence of anaemia (moderate: Hb<11g/dL and severe: Hb<6g/dL)

Mean haemoglobin concentration

Prevalence of drug resistance markers

Serology: prevalence of antibodies to CSP, MSP1 and AMA1

Costs of IPTc delivery: financial and economic costs to the provider

Incidence of serious adverse drug reactions

Baseline characteristics:

Characteristics of the 6 implementation zones will be tabulated, including DSS estimates of population size of children and including all age groups, bednet use recorded in each DSS round in children under 10 years and in older age groups, the mean distance to the nearest health facility, socio-economic status, religion and ethnicity.

Mortality rates:

The mortality rate ratio for the effect of IPTc will be estimated using Poisson regression with a gamma-distributed random effect for the cluster. An indicator variable for the intervention effect in each age group (3-59 months and 60-119 months) will be defined, set to 1 for each time period, age group and cluster when IPTc was implemented and set to 0 otherwise. A test of interaction will be done and combined estimate of intervention effect estimated if there is no evidence of an interaction. Age group (<3 months, 3-59 months, 60-119 months), and calendar year (2008,2009,2010) and zone will be included as covariates in the model. All deaths recorded in the DSS system will be included with person time obtained from the DSS. The DSS here refers to the DSS system set up for this project, and the Niakhar DSS. Person time includes all persons resident in the DSS area, from study start, date of birth or immigration, and censored when they left permanently or died. Temporary absence will not be accounted for as these dates are judged to be not sufficiently accurate and complete in the project DSS. Data updated to DSS round 4 will be included in the primary analysis.

Time periods:

Primary analysis of rates will be for the period from September 15 to December 15 each year. Secondary analysis will be done using data for the whole year.

Malaria incidence:

A similar model will be fitted to estimate the malaria incidence rate ratio. This will be done firstly for malaria cases that were confirmed by RDT or slide (i.e. cases treated for malaria at a health facility with a positive diagnostic test result), and secondly including all cases treated for malaria with or without confirmation. Two indicator variables for the intervention effect will be defined, the first one for direct effects* is set to 1 for each time period, age group and cluster when IPTc was implemented and 0 otherwise, the second for indirect effects is set to 1 in IPTc clusters for all age groups too old to receive the intervention, and 0 otherwise. For the direct effects, a test of interaction will be done and combined estimate of intervention effect estimated if there is no evidence of an interaction. Age group (0-59 months, 60-119 months, 10-20 years, 20-30 years, 30-40 years, 40-50 years, 50-60 years and 60+), and calendar year (2008,2009,2010) will be covariates in the model. The first age group is 0-59 months rather than 3-59 months because clinic records do not reliably note if the child was under 3 months of age. Malaria cases will be linked to the cluster (health post) based on the village of residence recorded in the register, not the health post where the case was treated. All cases at health huts, health posts, mission clinics, health centres and regional hospitals will be included. A list of the names of all villages and hamlets was prepared, 1247 in total, these were assigned a numeric code, this list was used when project staff visited health facilities to collect malaria data, to check if the village was within the project area, and assign a numeric code to the village of residence, or a code to indicate the village was outside the area.

*Note the model estimates indirect effects in the older age groups, while the effect in the intervention age groups represents total direct plus indirect effects. The direct effect of IPTc would be D=(T-I)/(1-I) where I is the indirect effect, if any, in older age groups, and T the total effect, observed in children.

The same DSS data as for mortality analysis were used for analysis of malaria incidence. For the rebound analysis, data updated to round 5 were included in the rebound analysis. Round 5 was incomplete (healthposts not visited in round 5: 39 DAROU SALAM, 41 GRAND MBOUR, 44 MBALLING, 45 MEDINE, 46 NGAPAROU, 47 NGUEKHOKH I, 48 NGUEKHOKH II, 49 POINTE SARENE,

50 SALY, 51 SANTESSOU, 54 VAREDO) for this reason population denominators for the rebound analysis were obtained by projection from the 2008-2010 data assuming a uniform annual growth rate of 3.6%.

Incidence the year after intervention will be compared between PSP and control areas in age groups that reflect the number of previous years of IPTc received, using a random effects poisson regression with adjustment for age effects.

Estimates of the coefficient of variation between clusters will be estimated for the main incidence rate endpoints (mortality and malaria), as √α where α is the variance of the exponentiated random effect reported by Stata in the xtpoisson output, from a model without covariates and from a model with covariates.

Analysis of cross-sectional surveys:

In December of 2008, 2009 and 2010, children in SMC and control areas were surveyed (children eligible for SMC in those years were included, i.e. in 2008, those who were aged 3-59 months on Septmebr 15^th^ of that year, and in 2009 and 2010, those that were aged 3 to 199 months on Septmebr 15^th^). Information was collected on receipt of IPTc, ITN ownership, ITN use and socio-economic status, and blood samples were taken for evaluation of parasitaemia, haemoglobin (in 2008 and 2009 only), and prevalence of markers of drug resistance and (in 2009) serological responses to *P. falciparum* antigens. Probability sampling was used with slightly different sampling designs in each year. In 2008, children in zones 1, 2 and 6 were surveyed. In each of the 27 health post areas in these zones, 5 villages were selected with probability proportional to size (PPS), in each selected village, compounds were listed in random order, and all eligible children in these compounds were included in the sample, continuing until the target number of children (20 per village) was exceeded. For health posts with one very large village and several small ones, the large village was treated as a stratum and the smaller villages as a second stratum and we sampled proportionately from each stratum, using systematic PPS to select the sample of 4 villages in the second stratum. Data were collected on a total of 2889 children (1099 in zone 1, 890 in zone 2 and 900 in zone 6). In 2009, a sample of 130 children was selected in each of the 54 health posts, (a total planned minimum sample size of 7020), households were selected in each health post by simple random sampling from the DSS database, and all the children who were normally resident in the household (have been resident for at least 6 months or, if resident for less than 6 months, then plan to remain in the area for at least a total of 6 months), and were eligible for an IPT dose in 2009 were included (i.e. children aged 4-123 months at the time of the survey). The sampling fraction of households therefore varied from health post to health post in order to yield a constant sample of 130 children per health post. In 2010, SMC was implemented in 45 of the 54 health posts and so the survey sample was weighted to over-sample the control areas. As the prevalence of parasitaemia was low the previous year, oversampling was also done in health post areas where the prevalence was expected to be greater, based on the incidence of malaria on the previous year. The list of settlements in the study area was sorted on zone and health post, to give an implicit stratification, and then 40 settlements were selected with probability proportional to size. A weighting to increase the number of samples from control areas (zone 6) and from areas that had more malaria was done by increasing the population size by a factor of 2 for settlements in zone 6, and for settlements in other areas that had a malaria incidence the previous year ≥3/1000, before systematic PPS sampling. 40 clusters were selected with at least 25 children to be sampled in each cluster. If a village had a population of less than 40 children in the SMC age range according to the DSS, the next village in the sorted list was added to the cluster after PPS selection. Households were listed in a random order and households were included for the survey if the cumulative number of children was 25 or less (stopping at the first household for which the cumulative number was 25 or more).

Response rates will be tabulated by healthpost and by PSP area. Reasons for non-response will be tabulated.

Survey analyses will use sampling weights calculated as the inverse of the sampling probabilities, with robust standard errors to take account of cluster sampling using svy commands in Stata.

Coverage:

Coverage of IPTc (the proportion with 0,1,2 and 3 treatments received) will be estimated using svy commands in Stata to weight the sample according to the survey design, using sampling weights calculated as the inverse of the probability of selection for a given household in each healthpost. The following measures of IPTc coverage will be calculated:

Overall coverage

Separate estimates of coverage for those with doses confirmed by their DSS card, and those without.

Number of courses received by age group. Of particular interest is the comparison of children over 5 years with those under 5.

Summary of reasons for missed doses.

Summary of problems with administration of the most recent dose.

Attendance at school and missed classes due to administration in November.

Coverage will also be analysed in relation to socioeconomic status and other factors:
SES obtained from household assets

Self-assessment of relative wealth, for comparison with the asset-based indicator

Equitability of coverage for IPT and ITNs

Distance of village to health facility (from GPS coordinates)

Caregiver’s level of education.

ITN Coverage:

Coverage indicators for ITNs (defined as intact or impregnated net) will be calculated:

Overall coverage defined as ownership and as use the previous night.

Separate estimates of coverage by age group (compare children over 5 years with under 5s).

Equitability of coverage for IPT and ITNs

Prevalence of parasitaemia:

For parasitaemia the following measures will be calculated:

Prevalence of *P.falciparum* asexual parasitaemia

Prevalence of *P.falciparum* gametocyte carriage

Prevalence by intervention area (PSP/non-PSP)

Prevalence according to ITN use

Prevalence of infection with *P. malariae* and *P.ovale*

The prevalence ratio between SMC and control areas will be estimated using survey poisson regression with a robust standard error to reflect the cluster sampling design. Interaction terms will be included to compare efficacy between age groups and to determine if there is any evidence of synergistic interaction between PSP and ITNs.

Prevalence of anaemia:

Prevalence will be calculated by intervention area (PSP/non-PSP) and according to ITN use (ITN defined as intact or impregnated net) and by SES group, for each age group. Interaction by age group will be assessed. Two endpoints will be considered, moderate anaemia (Hb<11 g/dL), and severe anaemia (Hb<6 g/dL). Analysis methods are similar to those for prevalence of parasitaemia.

Haemoglobin concentration:

Haemoglobin concentration will be analysed by normal regression, to estimate differences in mean Hb concentration in PSP and control areas, and by age group.

Drug resistance markers:

Prevalence of the following resistance-associated genotypes will be tabulated

pfdhfr-51, 59, 108 triple mutant (resistance to pyrimethamine)

pfdhps-437 and pfdhps-540 mutations (resistance to sulfadoxine)

Quadruple mutant (DHFR triple mutant plus DHPS 437 mutation)

pfcrt CVIET, pfmdr1 86Y, and pfmdr1 184F mutations associated with resistance to amodiaquine

The prevalence of resistant genotypes will be estimated in two ways. Firstly the proportion of slide-positive samples that are positive for the marker will be calculated. Secondly, the overall prevalence of the marker will be estimated, this is calculated as P_1_xP_2_ where P_1_ is the prevalence of asexual parasitaemia and P_2_ is the proportion of those who had parasitaemia, who were positive for the resistance genotype. A 95%CI for (P_1_xP_2_) is given by P_1_P_2_/EF to P_1_P_2_xEF where EF=exp{1.96x√[(1/P_1_)^2^V(P_1_)+ (1/P_2_)^2^V(P_2_)]}, where V(P_1_) is the variance of the survey estimate of the prevalence, obtained from the output from Stata svy commands, and V(P_2_) is the variance of the proportion of the n *P.falciparum*-positive samples that were positive for the mutation, [P_2_(1-P_2_)/n].

The prevalence (both measures) will be compared between PSP and non-PSP areas in each year of the study. To compare the overall prevalence measures, if the prevalence in PSP areas is a= P_1_P_2_ and in control areas b= P_3_P_4_, with V(a) and V(b) V(a)= P_2_^2^V(P_1_) +P_1_^2^V(P_2_) and V(b)= P_4_^2^V(P_3_) +P_3_^2^V(P_4)_, for the difference a-b, se(a-b)= √[V(a)+V(b)], so the 95%CI for the difference is (a-b)+/-1.96*se(a-b), and for the P-value we can use the z-test with z=(a-b)/se(a-b). For the risk ratio RR=a/b, need to work on a log scale again,

se(log(RR))=√ [(1/a2)V(a)+(1/b2)V(b)] = √[V(P_1_)/P_1_^2^ +V(P_2_)/ P_2_^2^ + V(P_3_)/P_3_^2^ +V(P_4_)/ P_4_^2^]

95%CI logRR+/-1.96xse(logRR), and z test, z=log(RR)/se(log(RR)).

Serology:

The prevalence of antibodies to apical membrane antigen (AMA)-1 and to merozoite surface protein 1 (MSP-1_19_) will be estimated in PSP and control areas in 2009, and by age group. The data will be used to investigate evidence for changes in transmission in the recent past by fitting a model for the seroconversion rate to the age-prevalence profile, in each district and in PSP and non-PSP areas. Seroconversion rates will be estimated using a superinfection model (S Bosomprah PhD thesis).

Adverse drug reactions:

Severe and serious cases of suspected adverse drug reactions reported through the pharmacovigilance system will be assessed for association with PSP drugs by a safety review panel. The incidence will be estimated in relation to the number of children treated. The incidence of all reported cases within 10 days of PSP rounds, from yellow forms collected from health facilities, will be tabulated by month and year and by age and gender, and by symptom type. Rates will be estimated in relation to the number of treatments administered.

1. ^*^ NA = Not Appropriate [↑](#footnote-ref-2)
2. ^1^ Centre d'études statistiques appliquées à la médecine [↑](#footnote-ref-3)
