# Supplementary material for: Effectiveness of Seasonal Malaria Chemoprevention in Children under Ten Years of Age in Senegal: A Stepped-Wedge Cluster-Randomised Trial
Source: PLoS Med. 2016 Nov 22;13(11):e1002175. doi: 10.1371/journal.pmed.1002175 (PMC5119693; doi:10.1371/journal.pmed.1002175)

S3 Text Methods

**Consent:** A series of meetings were held with the local government authorities and district health staff to explain the aims and activities of the project. In each of the three Departments of the project area (Bambey, Mbour and Fatick), the Prefet (the senior local government administrative officer of the Department) convened meetings of the Presidents of the Communites Rurales in his/her Department, at which the aims and activities of the project were explained using a pre-prepared information sheet for community leaders. At this meeting, further meetings were planned for village heads. The programme was then publicised through public meetings, local radio, and via village criers. On the first occasion when the intervention drugs were delivered through house to house visits, verbal consent to participate in the SMC programme was sought from the mother or carer of each eligible child by the community worker after explaining the programme using a standard script translated into the appropriate local language (Wolof or Serer). The information sheet mentioned the aims of the project and the potential side effects of the study drugs. Verbal consent or refusal was recorded in a register by the community worker. Consent was sought separately for participation in demographic surveillance. The plans for demographic surveillance were publicised through public meetings, local radio and village criers and in posters displayed in all health facilities. Households were visited to explain the aims and activities of the surveillance using a standard information sheet, verbal consent of the head of the household was sought and consent or refusal was recorded on a recording sheet by the interviewer. Consent was sought separately for participation in cross sectional surveys at the end of each year of the project. After publicising the survey in the community through meetings with community workers, the village head, and public meetings, selected households were visited to explain the survey, and signed consent was sought from the mother or carer of each eligible child after explaining the aims and procedures of the survey using a standard information sheet. Consent from community representatives and health staff (district medical officer and health post nurses) was sought to collect and store information about health facility attendances and hospital admissions in a database. The protocol was approved by the Ethics Committee of the London School of Hygiene&Tropical Medicine and by the Conseil national de recherche en santé (CNRS) in Senegal.

**Randomization:** The 54 health posts were randomized into 6 groups of 9, group 1 to receive intervention in year 1, groups 2 and 3 in year 2, and groups 4 and 5 in year 3 and group 6 in year 4. To ensure the 6 groups were comparable, the randomization was checked for balance, if the degree of imbalance was considered too large the randomization was repeated until an allocation with acceptable balance was achieved. The degree of balance was assessed with respect to the following five variables:

Distance from health post to health centre in km

Population size of the catchment area of the health post

Latitude and longitude of the health post

Performance of the health post as judged by the district medical officer (Satisfactory or Good) reflecting the degree of cooperation between the health post and the community.

Distance to the health centre was chosen as it is a proxy for access to health care; population size was included to ensure approximately equal number of children are included at each stage of implementation; latitude and longitude were included to allow for geographical variation in malaria incidence. The performance indicator was included, although it was unlikely to be related to primary outcomes, in order to balance the quality of delivery performance over the 4 years. District was not included explicitly since balancing on latitude and longitude should result in balance for district.

The total number of randomizations is:

$$\left(\begin{matrix} 54 \\ 9 \end{matrix}\right)\left(\begin{matrix} 45 \\ 9 \end{matrix}\right)\left(\begin{matrix} 36 \\ 9 \end{matrix}\right)\left(\begin{matrix} 27 \\ 9 \end{matrix}\right)\left(\begin{matrix} 18 \\ 9 \end{matrix}\right)/6!=1.40413\times10^{35}$$

(The no. of ways of putting 54 items into 6 groups of 9, divided by the number of ways to order the 6 groups).

For each variable in turn, different degrees of restriction were considered, and the proportion of randomizations that met the restriction was estimated (5 million randomizations were computed):

| Variable | Restriction* | % randomizations that met the restriction |
| --- | --- | --- |
| Distance from health centre | <2.5 km | 0.21% |
|  | <5km | 5.12% |
|  | <7.5km | 24.45% |
|  | <10km | 55.00% |
| Population size | <1000 | 0.021% |
|  | <2000 | 0.61% |
|  | <3000 | 3.92% |
|  | <4000 | 13.05% |
|  | <5000 | 29.56% |
| Longitude (^o^) | <0.05 | 0.150% |
|  | <0.1 | 3.66% |
|  | <0.15 | 18.43% |
| Latitude (^o^) | <0.05 | 0.249% |
|  | <0.1 | 5.86% |
|  | <0.15 | 26.79% |
| Number of posts performing well^#^ | 1 | 1.479% |
|  | 2 | 17.83% |
|  | 3 | 44.06% |
|  | 4 | 74.13% |
|  | 5 | 91.85% |

* largest difference in the mean value between randomized groups

^#^largest difference in the number of posts performing well between groups

Acceptable balance was defined as the following maximum allowed differences between randomized groups:

mean distance from health centre differs by <=7.5km

mean population size differs by <4000

mean deg longitude differs by <0.15

mean deg latitude differs by <0.15

no. of health posts performing well differs by <4

The proportion of randomizations that met this combined restriction was 0.19%. Therefore a total of 2.67x10^32^ permutations, met the combined restriction. With this large number of randomizations, standard statistical inference procedures should remain valid provided that the health posts are randomized independently. If the restriction applied results in two health posts always being randomized to the same group then standard inference procedures may not be valid. To check for independence, the probability of any pair being allocated together can be estimated, these probabilities should not be close to 0 or 1. The expected probability is 1/6=0.167, the estimated probabilities for each pair ranged from 0.029 to 0.24, with 98% of values between 0.091 and 0.21, the distribution is shown in the graph below:

**Survey design:** Surveys were undertaken in December to January of 2008, 2009 and 2010 to determine coverage of SMC in the area covered by health posts where SMC was delivered, and to compare the prevalence of parasitemia, and of molecular markers of resistance to SMC drugs, in SMC and non-SMC areas. The use of bednets was also recorded and caregivers were asked about household assets in order to estimate socio-economic status. In 2008 and 2009, haemoglobin concentration was measured. In 2009, samples were taken for serological analysis. All the children who were normally resident in the household (had been resident for at least 6 months or, if resident for less than 6 months, planned to remain in the area for at least a total of 6 months), and who were eligible for SMC (i.e. in 2008 those aged 3-59 months on September 15th, and in 2009 and 2010 those aged 3-119 months on September 15th) were included. A listing of all the eligible children in the selected households was generated from the DSS database, but interviewers were instructed to recruit all eligible children living in the household whether they were in the DSS listing or not, and regardless of whether they had received IPT. The surveys were therefore designed to give a representative estimate of the coverage of IPTc in the study area among the resident population at the time of the survey. The survey field teams comprised interviewers from the local DSS team, technicians from Dakar responsible for blood sampling, three supervisors (one in each district), and one field coordinator, supported by a technical team and data office in Dakar. A fieldworker manual was prepared, training sessions were held for field staff (existing DSS field interviewers familiar with the study area). Local community health workers were recruited in each health post area to help locate households. Questionnaires were piloted in the study area, changes were then made to some questions and a field plan drawn up for visiting each selected household on the basis of the estimated duration of each interview (about 15-20 minutes for each caregiver). Each survey was planned over 14 days, alternating health posts that had received IPT and posts that had not received IPT to avoid systematic bias in the timing of the interviews. Meetings were held with village heads to describe the survey and to seek their agreement. Then each selected household was visited by DSS interviewers to explain the aims and procedures of the survey to the mothers or carers of eligible children and to ask for signed consent for the interview and for taking a finger prick blood sample from the child. For each child, duplicate consent forms were signed and dated, one kept by the mother and the other kept by the field team. If the mother/carer agreed to the interview but refused blood sampling, this was indicated on both consent forms. One call-back visit was arranged if the mother or a child was absent. If the target number of children was not reached after all the selected households had been visited and call-backs completed, an additional listing of households, selected by simple random sampling from the same health post, was used. Sampling continued until the target sample size was reached. In each household visit, the interviewer asked about all children living in the household in the target age range. The mother/carer of each child was asked about their demographic characteristics, education, sources of income, household and personal assets, type of house and availability of electricity, telephone and water supply, and they were asked to assess their relative wealth compared with others in the community on a 5-point scale. For each child the duration of residence in the village was recorded, and the place where the child sleeps was inspected to record the type and condition of the net. In 2010, bednet use was recorded for all household members using a modified version of the MERG questionnaire. In areas where IPT had been delivered, IPT doses were recorded from the DSS card if available or from caregiver’s recall, and if scheduled doses were not received from the health worker, the mother was asked the reasons. Caregivers were asked about adherence to the unsupervised doses. A finger prick blood sample was taken from each child for haemoglobin measurement with a portable haemoglobinometer (in 2008 and 2009), to make blood spots on filter paper for parasitology (all years) and for serology (in 2009), and for thick smears for parasitaemia measurement (all years). Pre-printed labels bearing the sample number and a check digit were stuck onto the questionnaire, the filter paper and into a register where the date of the interview and Hb measurement were recorded. If the child had an Hb<7g/dL the mother was given a referral slip to take to the health post where the child would be given a course of iron tablets and severely anaemic children referred on to the district health centre for blood transfusion if required. At the end of each day each team leader checked completed questionnaires against the registers, labelling of filter papers. Questionnaires were checked for completeness by the district supervisor before being batched and sent to Dakar for data entry. In 2009, the largest survey, the survey field team comprised 21 interviewers from the DSS team, 24 technicians , organised in 6 teams with three supervisors, one field coordinator, supported by 9 vehicles, and coordinated by a technical team in Dakar.

The sampling design in each year was as follows. In 2008, health posts in zone 1, zone 2 and zone 6 were surveyed. In each of these 27 health posts (health post catchment areas), 5 villages were selected with probability proportional to size (PPS), in each selected village, compounds were listed in random order, and all eligible children in these compounds were included in the sample, continuing until the target number of children (20 per village) was exceeded, giving at least 100 children per health post. For health posts with one very large village and several small ones, systematic PPS sampling presents a problem, so the large village was treated as a stratum and the smaller villages as a second stratum and we sampled proportionately from each stratum, using systematic PPS to select the sample of 4 villages in the second stratum. Data were collected on a total of 2889 children (1099 in zone 1, 890 in zone 2 and 900 in zone 6). Three field teams (25 persons) were involved in data collection, one team per district, during the month of December and the beginning of January. In 2009, all 54 health post areas were surveyed. In each health post, households were selected by simple random sampling from the DSS database to give a sample of 130 children was selected in each health post, (a total planned minimum sample size of 7020). In 2010, SMC was being implemented in 45 of the 54 health posts, so to adequately represent control areas, the population in zone 6 was oversampled. In addition, as the prevalence of parasitaemia had been lower than expected in 2009, we also oversampled health post areas that had higher malaria incidence in 2009 in order to increase the yield of parasite-positive samples. Oversampling was done by increasing the population size by a factor of 2 for settlements in zone 6, and for settlements in other areas that had a malaria incidence the previous year ≥3/1000, before systematic PPS sampling. The corresponding weighting was then used in the analysis. The list of settlements in the study area was sorted on zone and health post, to give an implicit stratification, and then 40 settlements were selected with probability proportional to weighted size. At least 25 children were sampled in each cluster. If a village had a population of less than 40 children in the SMC age range according to the DSS, the next village in the sorted list was added to the cluster after PPS selection. Households were listed in a random order and households were included for the survey if the cumulative number of children was 25 or less (stopping at the first household for which the cumulative number was 25 or more).

**Microscopy quality control:** Slides were read by a team of experienced slide readers at IRD, Dakar. All slides found positive, were read by a second reader. If the two readings disagreed about slide positivity or there was a more than 2-fold difference in parasite density, a third reading was done. The geometric mean density of the closest readings was used if the slide was judged positive. If there was disagreement about positivity the third reading was definitive. For each slide reader, 10% of the slides declared negative were read by a second reader. If 10% or more of these slides were positive by the second reader, all slides by the first reader were re-read by a supervisor and those result considered definitive. A summary of the slides that were re-read is given in the table below.

|  | Positive second reading | Negative second reading | Total |
| --- | --- | --- | --- |
| Positive first reading | 174 | 2 | 176 |
| Negative first reading | 5 | 935 | 940 |
| Total | 179 | 937 | 1116 |

Slides negative at first reading and positive at second reading: 5 slides, 4 were positive only for gametocytes, and 1 concerned a slide with parasitemia less than 80 pf per microliter at first reading.

Slides positive at first reading and negative on second reading: these were 2 slides with parasitemia less than 160 pf per microliter at first reading. There were 2 cases where falciparum gametocytes were considered as *P.malariae* and this was corrected after quality control. For external quality control, a random selection of 600 slides were read independently at the Department of Parasitology, Universite Cheikh Anta Diop. There was disagreement over positivity in one slide.

**Entomological monitoring:** A study of populations of Anopheles and malaria transmission was conducted during the second year in the villages of Gate (Bambey Health district), Toucar (near Niakhar), Pointe Sarrène (Mbour Health district) and Darou Salam, a district of the town of Mbour. The study was conducted during the period of malaria transmission (July to October 2009) and during the dry season (April and November 2009). Field activities consisted of the collection of mosquitoes by night landing catches and indoor resting collections in bedrooms after spraying pyrethroids. A total of 192 man-nights of capture were made and 360 dwellings visited during the 24 monthly surveys conducted in 4 selected villages. The team comprised a coordinator, a senior supervisor, 2 technicians, 32 collectors (8 per village), and 4 supervisors (one per village), one student and a driver. Night landing catches were made during two consecutive nights (20h to 06h), inside (room) and outside (porch / courtyard) in two concessions in each selected village.

**Malaria surveillance:** Malaria was detected through passive case detection at health facilities. The study area was served by three regional hospitals, three district health centres, 54 government health posts, mission clinics, and by health huts. From 2007, health staff at health facilities were asked to follow the clinical algorithm shown below for diagnosis and treatment of persons presenting with a febrile illness (this algorithm was changed in 2015 to require an RDT for all febrile cases). Health huts employed RDTs from 2008 or 2009. A number of health huts were opened during the period of the study. The number of health huts (cases de santé) operating in each zone, during the study were as follows:

|  |  |  | Zone |  |  |  |  |
| --- | --- | --- | --- | --- | --- | --- | --- |
| Year | 1 | 2 | 3 | 4 | 5 | 6 | Total |
| 2008 | 8 | 2 | 10 | 9 | 15 | 3 | 47 |
| 2009 | 11 | 8 | 11 | 14 | 22 | 9 | 75 |
| 2010 | 11 | 9 | 11 | 14 | 22 | 9 | 76 |

Number of malaria cases and perentage confirmed by RDT during the study:

|  | Zone: 1 | 2 | 3 | 4 | 5 | 6 |
| --- | --- | --- | --- | --- | --- | --- |
| 2008 | 853 | 388 | 677 | 499 | 666 | 291 |
|  | 87.6% | 78.6% | 51.7% | 49.7% | 62.9% | 91.4% |
| 2009 | 201 | 117 | 162 | 253 | 218 | 113 |
|  | 98.5% | 94.0% | 100.0% | 96.0% | 99.1% | 97.3% |
| 2010 | 1170 | 465 | 546 | 833 | 780 | 852 |
|  | 99.6% | 95.7% | 99.6% | 88.5% | 98.6% | 95.4% |
| 2011 | 447 | 194 | 286 | 690 | 423 | 379 |
|  | 99.8% | 96.9% | 95.5% | 89.9% | 97.6% | 99.7% |

**Notes on study planning:** The aim of the study was to assess effectiveness of IPTc (Intermittent preventive treatment in children, now called SMC) delivered through district health services in three districts in Senegal. IPTc was highly effective in clinical trials but it was questioned whether it could be delivered on a large scale at reasonable cost, whether it would be acceptable to families, and there were concerns about safety of the drugs. It was thought that if an impact on mortality could be demonstrated this would strengthen the case for a policy recommendation. These considerations guided design and the sample size. A step-wedge design was chosen, trials had shown a clear benefit so this ruled out the use of a control group but as there was no established delivery channel implementation would need to be phased in gradually. It was thought that uncontrolled before-after designs would be difficult to interpret due to the variability in malaria year to year. Funding was secured for three years with a possibility of a fourth year if results were encouraging; it was thought that if early impact was dramatic it would be possible to attract further funding to continue the intervention after the study. Before the study, formative research was done to identify the most suitable methods for delivering the intervention, and a pilot implementation was done on a small scale to assess acceptability to communities and to health staff, and compliance, and to identify any important constraints, and to provide data to support a funding application for the main study. Parasitological diagnosis for malaria was introduced by the Senegalese MoH the same year as our project started. It was considered unreasonable to delay implementation for a year to get pre-intervention surveillance data since we had funding for the intervention, but this meant there was no pre-intervention data on confirmed malaria incidence in the first clusters to introduce intervention. Nevertheless it was felt appropriate to establish surveillance throughout the study area at the start, while implementing intervention more gradually, and it was useful to do so in order to maximise the pre-intervention data in each cluster, in view of the substantial variability in malaria incidence year to year and place to place. The original sample size calculations described in the protocol assumed more limited surveillance (in only parts of the pre-intervention areas) than was eventually adopted and were therefore in this sense conservative. Malaria cases were detected at health facilities, by the government nurse (at hospitals and health posts), at a mission clinic, and by community health workers (in villages with community case management for malaria). Confirmation was by RDT, or by microscopy. A small study was done to estimate sensitivity and specificity of the RDT compared to microscopy. Exit interviews were done at 12 health posts, interviewing all patients in a 2-week period, and the findings compared with entries in the clinic register to assess adherence to guidelines for testing and treatment and the completeness and accuracy of recording of malaria cases in health facility registers. Training workshops were held for health staff and close contact was maintained by supervisors who visited each health facility once every 1 to 3 months. Cases were linked to clusters (not to individuals), based on village of residence. Surveillance was maintained in all age groups, we could then estimate the indirect effect of the intervention in terms of a reduction in incidence in older age groups, as well as estimating the combined direct and indirect effect in the age groups targetted by the intervention. A pharmacovigilance system was established, based on the national system but strengthened through training, supervision, and use of SMS messaging. A simplified DSS was set up, with rounds of 10 months duration, and deaths were recorded through DSS rounds. Dates of doses were documented in registers and on a family-held record card. Usage and wastage of drugs was tallied. Coverage was estimated independently from surveys each year, sampling from the population resident at the end of the transmission season in each intervention area, to yield an overall estimate of coverage each year. Health officials and community leaders agreed to the idea of randomization by health post but asked that intervention progressed at the same rate in all three districts. The health post was the natural cluster, as each health post organises community programmes in their catchment area (median about 9000 people) and supervises a network of community health workers. Constrained randomization was used to avoid chance imbalance between intervention and control areas at each step, but we did not have good data on the key outcomes (confirmed malaria incidence, all cause deaths) by cluster, so the randomization had to be based on proxy variables (distance from river, distance from health centre, population size, number of villages). We also included an assessment of the performance and experience of the staff at the health post (on a 3-point scale) so as to avoid imbalance in ability to implement. A geographical constraint was included to avoid randomizations that resulted in clumps of intervention and control clusters. Partial re-randomization would have been possible at the end of year 1, using information about malaria incidence in each cluster in year 1 in the constraint, (in practice we didn't do this because the data were not ready in time). The randomization was kept secret, health staff knew their status for that year (implement or control) only at the start of that year. It was not easy to establish what other interventions were likely to take place in the study area during our trial, programmes are often organized at very short notice by different agencies. We knew the area was not one of those targetted for IRS. Large scale bednet distributions were conducted during the study, and mass administration of azithromycin was done in one district. We documented these interventions (timing, areas covered, coverage), we also recorded net-use through DSS rounds each year. These interventions tend to be delivered to whole districts so balancing the design by district reduced the scope for bias. During our study, WHO were reviewing evidence about SMC with a view to making a policy recommendation. Our data collection was modified in 2009 and 2010 to address some specific questions from the WHO consultative group relating to costs and safety, in 2010 detailed information on costs was obtained from all 46 facilities that implemented SMC (45 health posts and one mission clinic). A Technical expert group reviewed evidence about SMC including results from this study in 2011, SMC was recommended for high incidence areas, our study area fell outside these recommendations but SMC is now being implemented in the south of the country where the burden is higher. We did not do a case control study of efficacy within the trial but with hindsight this would have been useful, to measure the efficacy of SMC treatments.


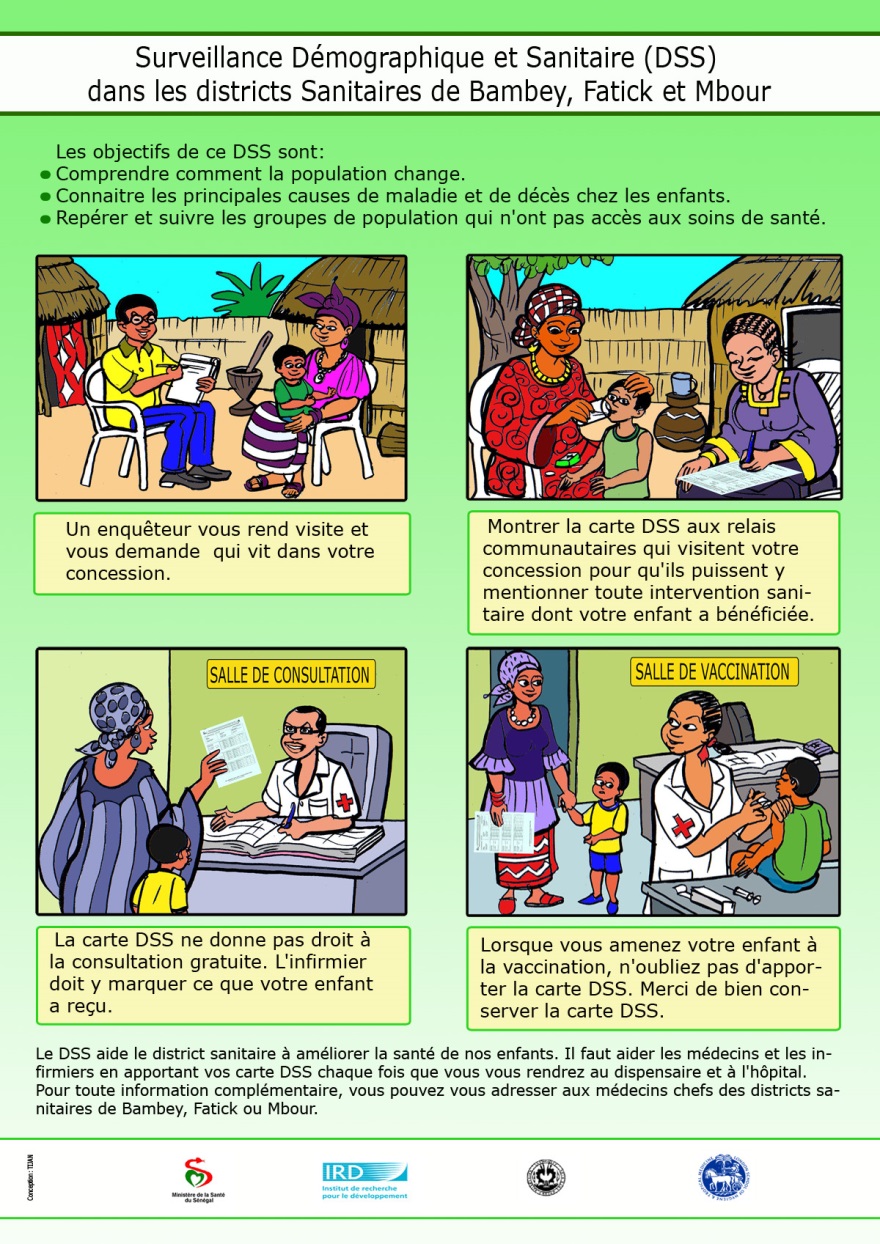

Supplement: S3 Text — (DOCX) [file pmed.1002175.s016.docx]
